# The dynamics of RAD51 foci formation and elongation in living human cells

**DOI:** 10.1093/nar/gkag863

**Published:** 2026-09-11

**Authors:** Anoek Friskes, Melanie Snoek, Lisa Koob, Roel Oldenkamp, Bram van den Broek, Leila Nahidiazar, Lukas Frank, Amalie E Dick, Marjolijn Mertz, Rolf Harkes, Benjamin D Rowland, Kees Jalink, René H Medema

**Affiliations:** Division of Cell Biology, The Netherlands Cancer Institute, Amsterdam 1066 CX, The Netherlands; Oncode Institute, Plesmanlaan 121, Amsterdam 1066 CX, The Netherlands; Division of Cell Biology, The Netherlands Cancer Institute, Amsterdam 1066 CX, The Netherlands; Oncode Institute, Plesmanlaan 121, Amsterdam 1066 CX, The Netherlands; Division of Cell Biology, The Netherlands Cancer Institute, Amsterdam 1066 CX, The Netherlands; Oncode Institute, Plesmanlaan 121, Amsterdam 1066 CX, The Netherlands; Division of Cell Biology, The Netherlands Cancer Institute, Amsterdam 1066 CX, The Netherlands; Bioimaging facility, The Netherlands Cancer Institute, Amsterdam 1066 CX, The Netherlands; Division of Cell Biology, The Netherlands Cancer Institute, Amsterdam 1066 CX, The Netherlands; Oncode Institute, Plesmanlaan 121, Amsterdam 1066 CX, The Netherlands; Bioimaging facility, The Netherlands Cancer Institute, Amsterdam 1066 CX, The Netherlands; Bioimaging facility, The Netherlands Cancer Institute, Amsterdam 1066 CX, The Netherlands; Bioimaging facility, The Netherlands Cancer Institute, Amsterdam 1066 CX, The Netherlands; Division of Cell Biology, The Netherlands Cancer Institute, Amsterdam 1066 CX, The Netherlands; Division of Cell Biology, The Netherlands Cancer Institute, Amsterdam 1066 CX, The Netherlands; Division of Cell Biology, The Netherlands Cancer Institute, Amsterdam 1066 CX, The Netherlands; Oncode Institute, Plesmanlaan 121, Amsterdam 1066 CX, The Netherlands

## Abstract

Homologous recombination is a DNA repair process that requires binding of RAD51 to ssDNA at the break site. This facilitates the search for a homologous repair template on the sister chromatid, or on the homologous chromosome. How broken DNA ends loaded with RAD51 filaments are brought toward their repair template in the crowded 3D genome is currently poorly understood. This is largely due to a lack of tools to visualize homology search in living human cells. Here, we show that RAD51 and MND1, two proteins operating in homology search, become visible in long, extended structures several hours after double-stranded break formation. Using GFP-MND1 we capture these elongated foci in living human cells and reveal their highly dynamic nature as they traverse the nuclear space and gradually disassemble. We show that resolution of these structures depends on RAD54L, known for its role in RAD51-driven homology search. In addition, we find that loss of cohesin inhibits their resolution, in accordance with a role for cohesin in homology search. Thus, our data suggest that these elongated foci are visible intermediates of an active DNA repair process, and that GFP-MND1 is a powerful tool to study the dynamics of homology search in living human cells.

## Graphical Abstract

**Graphical Abstract:**
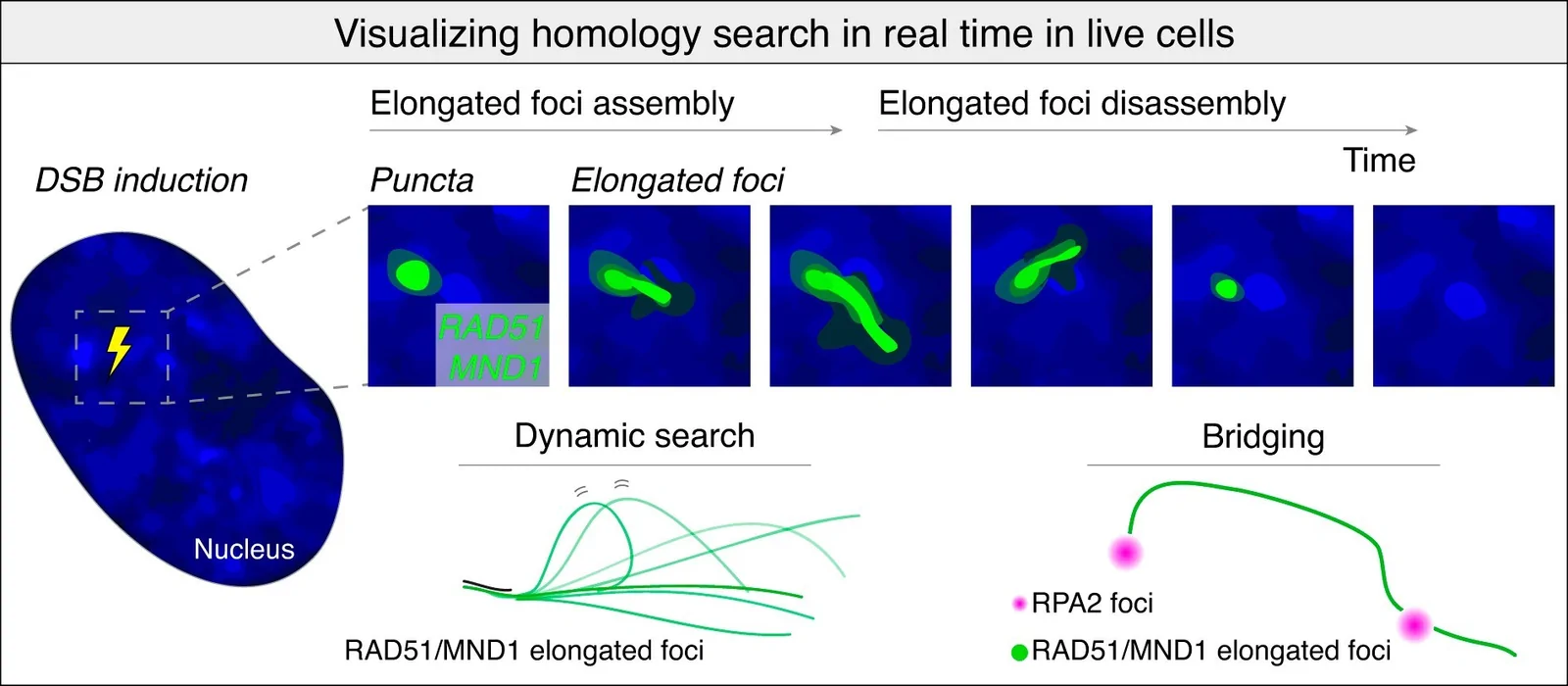

## Introduction

One of the principal pathways employed by cells to faithfully repair DNA double-strand breaks (DSBs) is homologous recombination (HR) [1]. HR is important for error-free repair in somatic cells, and for the pairing of homologous chromosomes in meiosis. HR relies on the use of an intact homologous DNA sequence as a template for accurate repair [2]. To engage in HR, the 5′ ends on either side of the DNA double-stranded break are first resected by nucleases such as MRE11, EXO1, and DNA2 to generate extensive 3′ ssDNA overhangs [3]. Once ssDNA is generated, it is rapidly coated and stabilized by the protein complex RPA (replication protein A), which is subsequently replaced by the recombinase RAD51 [3]. RAD51 forms a nucleoprotein that guides the coated ssDNA along the double-stranded DNA to identify the homologous sequence [4]. RAD51 nucleoprotein filament formation is an essential step in HR, and a failure to replace RPA with RAD51 can create intermediates that cannot be properly processed and that can become detrimental to cells [5].

The distance that the nucleoprotein filament needs to cover to find its proper template will likely affect the efficacy of the homology search process. In human somatic cells, HR is (most) active in S/G2 cells, when a sister chromatid is available in relatively close proximity. Recent studies found that sister loci are separated by DNA looping [6], causing them to spatially separate over distances up to 1.5 micron [7]. In meiotic cells, however, the nucleoprotein filament needs to avoid the sister template and instead find the right template on the homologous chromosome that is not necessarily close by [8]. In addition, interhomolog recombination does also occur in somatic cells [9], which could necessitate extensive filament expansion to span the distance between distinct chromosome territories. In case the distance between a DNA break and its homologous template is large, the search for the correct repair template in the vast and crowded nuclear space can become a challenge. This challenge comes with the risk of selecting an incorrect donor sequence, thereby promoting genomic instability [9, 10].

Already decades ago, studies conducted in bacteria showed that the bacterial RAD51 homolog RecA forms elongated structures during homology search and strand invasion at sites of DSBs [11–14]. In line with this, various subsequent genetic*, in vitro*, structural, and molecular studies indicated that RAD51 can also form nucleoprotein filaments [15–17]. While most studies report the formation of (spherical) RAD51 foci following irradiation-induced DNA damage, some have shown that extended structures positive for RAD51 can also be observed at higher resolution, both in somatic cells and oocytes [18, 19]. Elegant single molecule localization microscopy experiments in HeLa cells have demonstrated that RAD51 clusters at DNA damage foci and progressively extends into elongated RAD51-positive structures over time [18]. These extended structures undergo topological changes and reach a maximal length ∼3–5 h after damage induction, suggesting that they represent RAD51 nucleoprotein filaments engaged in homology search [18]. This concept was more recently supported by live-cell imaging of endogenously GFP-tagged Rad51 in yeast, demonstrating highly dynamic [20] RAD51-coated structures undergoing rounds of extension and compaction [20]. These structures were referred to as “filaments”, but this should not be confused with the term “RAD51 filament” that refers to the helical nucleoprotein structure that RAD51 forms on DNA. RAD51 nucleoprotein filaments are likely present in the early foci (or puncta, see below), as well as in the extended structures. We therefore refer to the extended structures as “elongated foci”. These elongated foci can span the entire yeast nucleus and establish contact with the template sequence. Thus far, real-time dynamics of homology search in human cells have not been visualized.

In the current study, we use quantitative 3D super-resolution (SR) imaging of fixed cells, complemented with high-resolution live-cell imaging to investigate the homology search process. We find that puncta of RAD51, as well as MND1, that appear early after double-stranded break formation, stretch into elongated structures as repair proceeds. These structures can span very large distances and live-cell imaging reveals that they dynamically explore the nuclear space and can resolve over time. Notably, this resolution depends on RAD54L and cohesin, both proteins with a previously established role in HR. We propose that the formation of elongated foci reflects a crucial step in HR when a nearby template is not available and that GFP-MND1 represents a powerful tool to study the dynamics of homology search in human cells.

## Materials and methods

### Cell culture and generation of CRISPR–Cas9 edited cell lines

RPE-1 cells were cultured in Dulbecco’s modified Eagle’s medium/Nutrient Mixture F12 (DMEM/F12, Gibco) supplemented with 6% fetal bovine serum (FBS, Sigma), 1% GlutaMAX (Gibco), and 100 U·ml^−1^ penicillin–streptomycin. HAP1 (Brummelkamp Laboratory, Amsterdam, The Netherlands) cells were cultured in Iscove’s modified Dulbecco’s medium (IMDM, Gibco) supplemented with 12% FBS, 1% GlutaMAX (Gibco), and 100 U·ml^−1^ penicillin–streptomycin. U2OS were cultured in DMEM (Gibco) supplemented with 6% FBS, 1% GlutaMAX (Gibco), and 100 U·ml^−1^ penicillin–streptomycin. HCT116 cells were cultured in RPMI 1640 (Gibco) supplemented with 12% FCS, 1% GlutaMAX (Gibco), 1% HEPES (Gibco), 1% MEM nonessential amino acids (Gibco, Waltham, USA), and 100 U·ml^−1^ penicillin–streptomycin. All cell lines were tested negatively for mycoplasma contamination before experiments were performed.

For gene-editing experiments, 250 000 cells were plated in a six-well dish, the next day, cells were transfected with a synthesized guide RNA (IDT) and the corresponding *trans*-activating CRISPR (tracr) RNA (IDT) that were mixed in equimolar concentrations to create a final duplex of 20 nM in OptiMEM (Gibco). The mixture was then transfected using RNAiMAX (Invitrogen) in Cas9 expressing RPE-1 cells (Dox/Shield-1 induced) or HAP1 pcW-Cas9 cells. Twenty-four hours later, the media was refreshed and cells were grown for an additional 48 h. Single cell clones were derived by limiting dilution. Successful editing of selected clones was verified by using TIDE analysis [21] using the Sanger sequencing of the polymerase chain reaction (PCR) products. Loss of the targeted protein was also assessed by immunoblotting.

### Western blot analysis

Western blot analysis was conducted as previously described [22]. Briefly, proteins were separated by sodium dodecyl sulfate–polyacrylamide gel electrophoresis and transferred onto nitrocellulose membranes. The membranes were then blocked in 5% bovine serum albumin (BSA) or 5% milk in TBS-T and incubated with primary antibodies overnight at 4°C, with antibody dilutions specified below. Following this, secondary antibodies were applied for 1 h at room temperature. Protein visualization was achieved using enhanced chemiluminescence (ECL; GE Healthcare, Chicago, USA).

### Antibodies and chemicals

The following primary antibodies were used in this study: anti-H2AX ser139 (yH2AX, 05-636, Upstate, Burlington, USA, 1: 500 IF, 1:1000 WB), anti-RAD51 (ab63801, Abcam, Cambridge, UK, 1:500 IF), anti-GFP (11814460001, Roche, Basel, Switzerland, 1:1000 WB/IF), anti-Rad54 (F-11): (sc-374598, Santa Cruz, Dallas, USA, 1 : 1000 WB), and anti-HSP90 (sc7947, Santa Cruz, Dallas, USA, 1 : 1000 WB). The following secondary antibodies were used for western blot experiments: peroxidase-conjugated goat anti-rabbit (P448, DAKO, Santa Clara, USA, 1:1000) and goat anti-mouse (P0447, DAKO, Santa Clara, USA, 1:1000). Secondary antibodies used for immunofluorescence were goat anti-mouse-Alexa 488 (A11029, Mol Probes, Waltham, USA, 1:1000) and goat anti-rabbit-Alexa 568 (A11011, Mol Probes, Waltham, USA, 1:1000). Chemicals used in this study are neocarzinostatin (NCS, N9162, Sigma, Waltham, USA), Halo ligand Janilla fluor (Promega), and 5-Ph-IAA (BioAcademia, Japan, #30-003).

### siRNA and crRNA transfections

For small interfering RNA (siRNA) transfections, RNAiMAX (Invitrogen, Waltham, USA) was used according to the manufacturer’s guidelines. The following siRNAs were employed in this study: non-targeting siNT and siRAD54L smart-pool all obtained from Dharmacon (Cambridge, UK). For CRISPR RNA (crRNA) transfections, the crRNAs were designed to target specific exons or sequences. These crRNAs were transfected using RNAiMAX in OptiMEM (Gibco, Waltham, USA). The transfection procedure involved incubating 20 nM crRNA and tracrRNA with RNAiMAX for 20 min before adding the mixture to the cells. Indel generation in the RAD54L locus was confirmed using TIDE analysis of PCR products.

### Ionizing radiation and clonogenic outgrowth

Cells were subjected to ionizing radiation using a Gammacell Exactor (Best Theratronics, Ottawa, Canada) equipped with a 137Cs source. To assess the sensitivity of various cell lines to γ-irradiation, a low density of cells was plated per well. These cells were then treated with different doses of irradiation and allowed to grow into single colonies over a period of 6 days. After this incubation period, the number of colonies formed was counted and normalized to the number of colonies in the untreated control condition.

### IncuCyte-based proliferation and caspase-3/7 activity assays

IncuCyte assays were performed in triplicate, using black-walled 96-well plates (Greiner, 655090). Cells were plated at a low density and allowed attachment overnight. Plates were then placed in the IncuCyte ZOOM (Essen Bioscience), which imaged the cells every 4 h. IncuCyte caspase-3/7 green apoptosis assay reagent (Essen Bioscience, 4440) was added to the culture medium. The next day, HAP1 cells were either left untreated or irradiated (2 Gy) right before imaging. Phase-contrast images were collected and analyzed to detect cell proliferation based on confluence. Green fluorescent images were also collected and analyzed (by dividing the detected green fluorescence confluence by the phase-contrast confluence) to calculate caspase-3/7 activity as a proxy for apoptotic cells.

### Constructs

pCW_HALO-MND1 plasmid was cloned by PCR amplification of MND1 from a construct previously generated [22] using the following primers:

fwd 5′-AGCGCTTCGGCTGGAGGAAGTGGTtcaaagaaaaaaggactgagtgca-3′ rev 5′-AAAAGGCGCAACCCCAACCCCACGCGTttagtctatgtagtcaaagtcttctggaattcc-3′ and HALO from plasmid DNA with the following primers: fwd1 5′-TCGCCTGGAGAATTGGatggcagaaatcggtactggc-3′ rev1 5′-CAGCCGAAGCGCTGCCGGAAATCTCGAGCGTAc-3′;

fwd2 5′-GCTCTACAAAAGCGCTTCGGCTGGAGGAAGTGGTtcaaagaaaaaaggactgagtgca-3′ rev2 5′-AAAAGGCGCAACCCCAACCCCACGCGTttagtctatgtagtcaaagtcttctggaattcc-3′. The PCR products were ligated into a cut-open (AgeI, EcoRI) pCW vector with BFP-T2A-Blast selection marker by Gibson ligation. Plasmids were then lentivirally transduced into RPE-1 and HCT116 cells and selected with blasticidin.

### Lentiviral transductions

HEK293T cells (5 × 106) were plated in a 10-cm dish the day before they were transfected with packaging plasmids: 4 μg of pMD2.G (Addgene,12260), 4 μg of psPAX-2 (Addgene,12259), and 4 μg of the target vectors using PEI. Viral supernatant was collected 48 h after transfection, filtered (0.45 μm syringe), and used for transduction in the presence of polybrene (8 μg/ml; Sigma–Aldrich). To select out positive transduced cells, cells were treated with blasticidin (15 μg/ml) until all non-transduced cells died.

### Immunofluorescence

Cells were seeded on coverslips (Thermo Fisher Scientific) and treated as indicated in the figure legends. To stain for GFP (1181440001, Roche), RAD51 (anti-RAD51, ab63801 Abcam), or yH2AX (yH2AX, 05-636 Upstate), (RPA2, Calbiochem), (BRCA2, Calbiochem) foci in cells, cells were fixed and permeabilizated with 4% PFA and 0.5% Triton X-100 in phosphate buffered saline (PBS), slides were incubated with primary antibodies in IF buffer for 1 h at room temperature. Cells were then washed three times with PBS-T and subsequently blocked in PBS supplemented with 0.1% Tween-20 (PBS-T) and 5% BSA for 30 min. Primary antibody incubation was performed overnight at 4°C (antibodies and dilutions stated below). Coverslips are washed with BSA in PBS-T, and secondary antibody (Invitrogen) and DAPI (2.5 μg/ml; Sigma–Aldrich) incubation is performed at room temperature for 1 h. Then coverslips were washed with PBS. EdU was stained by incubation in EdU staining buffer (100 mM Tris–HCl, pH 8.5, 1 mM CuSO_4_), with 100 mM ascorbic acid and AF-647 azide (Invitrogen, 1:1000) for 30 min at room temperature. Then, coverslips were washed again and mounted with Prolong Gold (Invitrogen,) and stored at 4°C.

All fluorescent images were acquired on a THUNDER Imager (Leica Microsystems), equipped with 63X/1.40 oil objective with HC PL APO objective and deep-cooled 4.2 MP sCMOS camera and processed with thundering. Imaged were acquired in three dimensions with a z-step of 500 nm. For optimal visualization the images are displayed as maximum intensity projection of the individual z-sections. For higher resolution, images were acquired on a Zeiss LSM 980 with Airyscan2 and 63X/1.4–0.6 oil objective in SR mode. After acquisition images were processed with ZEN software (Zeiss), applying the same processing strength per experiment. Using Imaris (Oxford Instruments), RAD51 structures were segmented with the surface algorithm and filtered by sphericity and longest axis using the bounding box measurement. Quantification of DNA damage-induced foci was performed in Fiji using CLIJ2/x libraries [23] with an in-house designed ImageJ macro (https://github.com/Bioimaging-NKI/Foci-analyzer), as described [22]. Briefly, nuclei are segmented in the DAPI channel using StarDist [24]. Total DAPI intensity is used to separate G1 and G2 phase cells. DNA damage foci are detected by applying marker-controlled watershed on local maxima in the foci channels, after which foci intensities and numbers are quantified per cell.

### 2dSTORM imaging and sample preparation

Samples were prepared as detailed described before [25]. In brief, RPE-1 HALO-53BP1 GFP-MND1 cells were seeded on an ultra-clean coverslip. As described before, immunofluorescence staining was performed where after blocking with 5% BSA (Serva, Heidelberg, Germany) for 1 h, cells were stained as follows. Cells were incubated with mouse anti-GFP (11814460001, Roche). Subsequently all the cells were incubated with a goat anti-mouse antibody (Alexa-488, Thermo Fisher Scientific) and DAPI to stain the DNA at a final concentration of 0.01 mg/ml for 1 h. The coverslip was mounted in a holder (Chamlide CMB mounting ring CM-B25-1, Live Cell Instrument, Seoul, South Korea) filled with 500 μl of OxEA imaging buffer. The OxEA buffer contains 50 mM β-mercaptoethylamine hydrochloride (MEA, Sigma–Aldrich), 3% (v/v) OxyFlour™ (Oxyrase Inc., Mansfield, Ohio, USA), and 20% (v/v) of sodium dl-lactate solution (L1375, Sigma–Aldrich) in PBS, pH adjusted to 8–8.5 with NaOH as described before [26]. Samples were imaged with the 488 nm/150 mW laser on a Leica SR-GSD microscope (Leica Microsystems, Wetzlar, Germany) equipped with an EMCCD camera (Ixon DU-897, Andor). We used a 160× oil immersion-dedicated SR objective. Around 4000 frames were collected at 100 Hz with image size of 180 × 180 pixels. The data were analyzed with the ImageJ ThunderSTORM plugin [27], after background-correcting with a running-median background subtraction [28] using an inhouse developed ImageJ plugin (https://github.com/Jalink-lab/Temporal-Median-Background-Subtraction).

### Pipeline for high-content analysis of foci dynamics

To quantify RAD51 object area and length, RAD51 structures were measured with Ilastik (v.1.3.2), using a pixel and an object classification. First, several FIJI in-house designed macros (https://zenodo.org/records/20677613) were used to crop individual nuclei using the DAPI channel and semi-automatically segment RAD51 objects in 3D. The obtained label images were overlayed with raw data, allowing adjusting segmentation parameters. Label images were maximum intensity z-projected and used as input for Ilastik, where object features (ObjectArea and total skeleton length) were used for classification.

### Live cell imaging analysis

Timelapse images are first deconvolved using Huygens Professional. The macro Split_multiseries_files_and_z-project.ijm then reads .czi files with Bio-Formats and creates maximum intensity z-projection timelapse images. The macro: Preprocess_segment_track_extract_and_register_nuclei.ijm performs preprocessing, nuclei segmentation, tracking, and image registration to stabilize the movement of individual nuclei. A median subtraction is applied to remove the static background while preserving moving nuclei. Since no dedicated nuclei marker is available, all channels are summed to maximize photon counts from the foci channels; outliers (the foci) are then removed and nuclei are segmented with StarDist across all time frames. TrackMate is used on the resulting label map to produce a tracked label map, from which single nuclei tracks are extracted from the original timelapses. These are then registered using a rigid body transformation to correct for nuclei rotation and drift (HyperStackReg plugin). For each nucleus, the registered tracked images on a black background are saved alongside a full-image tracked label map. The macro Overlay_cell_tracking_labelmap_for_timelapse_3ch.ijm overlays nucleus outlines from the tracked label map together with track numbers on the timelapse images, enabling visual inspection of tracking results and selection of nuclei for downstream analysis. Zeropad_tracks_and_combine_registration.ijm corrects TrackMate file naming (e.g. 1 → 001) and combines tracked and registered single-nucleus timelapse images for visualization of registration results. GFP-MND1 structures are segmented with Segment_with_labkit_and_merge.ijm, which runs an existing LabKit pixel classifier to produce a binary timelapse or 3D z-stack mask depending on the input, then merges the segmented mask as a red channel with the selected segmentation channel of the original image in grayscale. For timelapse images, TrackMate is subsequently run on the LabKit mask to follow individual foci or structures within each nucleus. Connected component analysis is then performed on the LabKit segmentation masks to create 3D object maps, with maximum intensity projections generated for 2D analysis. Finally, Skeletonize_and_measure_labels.ijm skeletonizes the label images (2D or 3D) and measures length and shape features using CLIJ and MorphoLibJ. Because the width of the structures prevents skeletons from extending fully to the ends, and because roundish structures can yield very small or single-pixel skeleton lengths, a correction is applied: for each endpoint, the shortest distance to the label edge is added to the skeleton length, and for structures with only one endpoint (near-circles or near-spheres), this distance is added twice. The corrected value is reported as Totallength. All macros are deposited at https://zenodo.org/records/20677613.

### Quantification of different focus topologies

For quantification of RAD51 focus topologies, cells were imaged on an LSM980 microscope using AiryScan mode at 6 and 16 h after irradiation. Using object classification in Ilastik, the percentages of different RAD51 focus topologies were quantified. First, representative images were used for manual annotation and training. For RAD51 structure quantification, the same FIJI macros were used before and 3D focus stacks (50 × 50 pixels) were input and segmentation maps, generated with ImageJ were used as input. Size in pixels, mean intensity, and total intensity were used for classification and output. To ensure confident structure assignment, a probability threshold was applied: a focus was assigned to a given category only if its probability score for that category exceeded the sum of its probability scores for all other categories combined. Foci that did not meet this criterion were excluded from the analysis. For quantification of GFP-MND1 focus topologies, cells were imaged by spinning disk confocal microscopy at 6 and 16 h after irradiation. Individual GFP-MND1 structures were classified by visual inspection into the following categories: Puncta and elongated foci. Classification was performed blind to experimental condition on maximum-projected images, and each focus was scored independently. Only unambiguous structures were included in the analysis.

### Live-cell microscopy

For live-cell microscopy, RPE-1 HALO-53BP1 GFP-MND1 cells were seeded in a Lab-Tek II chamber coverglass (Thermofisher, 8-well) and kept overnight. Live-cell imaging was performed on a Andor Dragonfly spinning-disk confocal microscope system equipped with an Andor Dragonfly system on an inverted Leica DMI8 microscope with an incubation chamber (OKOlab) for temperature (37°C) and CO_2_ control (5%). Images were acquired with a Leica 63×/1.47 NA oil immersion objective and a sCMOS Zyla 4.2 (Andor). Images were acquired every 5  min for a time-course of 24 h in three dimensions with z-steps spaced out by 500 nm. The max-projections were processed in FIJI applying a maximum intensity z projection. It is important to note that the above-described measures to enable three color live cell imaging come at the expense of detailed z-depth.

### DNA-FISH

DNA-FISH (fluorescence *in situ* hybridization) staining was performed in two sequential hybridization steps. First, unlabeled oligonucleotide probe pools tiling the target sequence were hybridized (primary hybridization). In the second step, fluorophore-conjugated probes were annealed to non-complementary tail sequences present in the primary probes (secondary hybridization). For DNA-FISH, treated or untreated cells were seeded in eight-well Nunc Lab-Tek II chambers (Thermo Fisher, cat. no. 155409PK) 1 day before staining. Cells were rinsed twice with PBS and fixed for 15 min in 4% formaldehyde in PBS at RT. After two additional PBS rinses, samples were permeabilized in 0.5% Triton X-100 in PBS containing 100 µg/ml RNase A (Thermo Fisher, cat. no. EN0531) for 15 min at RT, followed by denaturation in 0.1 M HCl for 15 min. The sample was then equilibrated for 5 min in 2× SSC buffer and then for at least 30 min in 2× SSC/50% (v/v) formamide before proceeding with the primary hybridization step. For hybridization, the cells were heated at 80°C for 7 min in hybridization mix containing 3.3 nM primary probe pool diluted in 15% (w/v) dextran sulfate (Merck, cat. no. 265152–50GM), 1 mg/ml BSA (Invitrogen, cat. no. AM2618), 42.5% (v/v) formamide (Sigma–Aldrich, cat. no. 344206-100ML), 0.05 µg/µl human Cot-1 DNA (Invitrogen, cat. no. 15279011), and 0.2 µg/µl yeast tRNA (Invitrogen, cat. no. AM7119) in 2× SSC buffer and then incubated for at least 16 h at 37°C in a humidified chamber. The next day, samples were rinsed once in 2× SSC buffer, followed by three washes each in 1× SSC buffer and 0.1× SSC buffer at 42°C. After equilibration in 2× SSC for 5–10 min, secondary probe hybridization was carried out for 1 h at 37°C in 2× SSC/10% (v/v) formamide and at secondary probe concentration of 10 nM. Samples were then incubated for 5 min in 2× SSC buffer containing 5 µM DAPI, rinsed three times in 2× SSC buffer and then stored in 4× SSC buffer at 4°C until imaging. Tiling oligonucleotide probe pools (primary probes) targeting the human DHFR gene (112 unique probes, NCBI gene ID 1719) were designed using the OligoMiner App39 followed by manual curation using BLAT search (UCSC genome browser). Primary probes (unconjugated oPools) and fluorophore-conjugated secondary probes (5′ATTO565 conjugated) were synthesized by Integrated DNA Technologies (IDT).

## Results

### RAD51 forms elongated foci at sites of DNA damage

**Figure 1.**
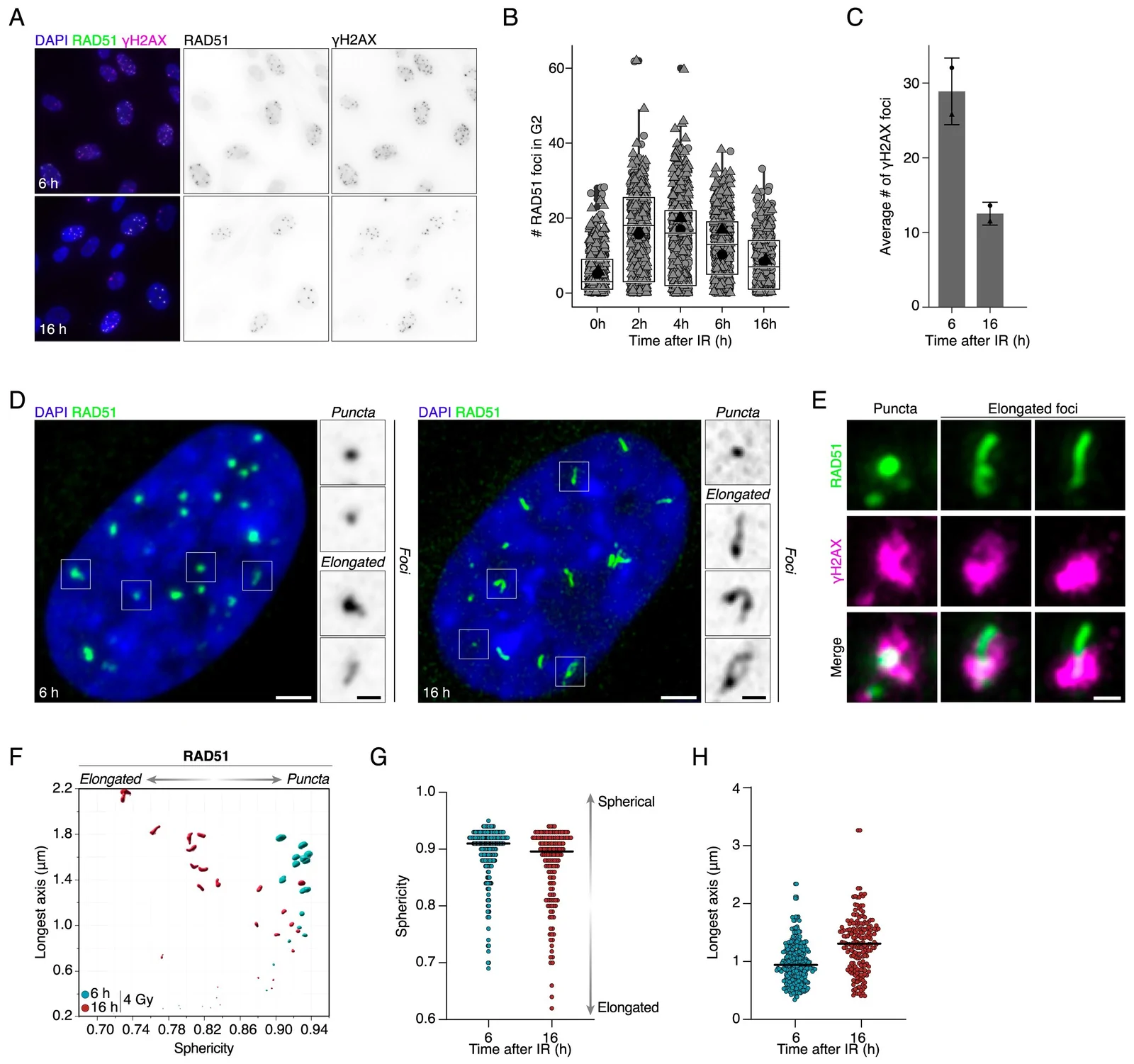
Elongation of RAD51 foci at sites of DNA damage. (**A**) RAD51 (middle panels) and γH2AX (right panels) foci in RPE-1 HALO-53BP1 cells exposed to irradiation [4 Gy, 6 h (upper panels) and 16 h (lower panels)] obtained using wide-field fluorescence microscopy. Quantification of the amount of RAD51- (**B**) or γH2AX-foci (**C**) at the indication time points after irradiation (4 Gy); gray dots representing individual cells, black dot/triangle indicating the average foci no. within each replicate experiment (*N* = 3). (**D**) RAD51 foci (puncta and elongated foci) in RPE-1 cells imaged using SR microscopy (blue; DAPI). Cells were irradiated with 4 Gy and fixed 6 (left panel) or 16 h (right panel) later. Scale bar, 2 μm. Insets, 0.5 μm. (**E**) Puncta and elongated foci stained for RAD51 (top panels) and γH2AX (middle panels) in RPE-1 cells resolved by SR. Cells were irradiated with 4 Gy and fixed 16 h later. Scale bar, 1 μm. (**F**) Left plot: Sphericity versus longest axis of RAD51 foci measured in a single cell in 3D, 6 or 16 h after irradiation (4 Gy). Actual structures are plotted in the graph. Distribution of the sphericity (**G**) and longest axis (**H**) of all RAD51 foci measured across multiple cells. The higher the value for sphericity, the more spherical (puncta) a focus is; the lower the value, the more elongated the focus is. Data were obtained through Imaris.

To explore RAD51 nucleoprotein filament dynamics in non-transformed human cells, we used immortalized RPE-1 cells. In these cells, RAD51 foci formation is observed within 2 h after irradiation (IR, 4 Gy) and remains high at 4 and 6 h after damage induction (Fig. 1A and B). At 16 h after damage induction, a significant fraction of the RAD51 and γH2AX foci has been resolved (Fig. 1A–C), indicating that part of the breaks that engage in RAD51-dependent repair are repaired between 6 and 16 h. Using SR microscopy, we found that at the 6 and 16 h time points, a fraction of RAD51-positive structures appeared as small dots, while others appeared as elongated foci of variable lengths and topology (Fig. 1D). For simplicity, we refer to the dot-like foci as “puncta”, and the extended assemblies as “elongated foci” throughout this manuscript. The dual appearance of RAD51 in puncta and elongated foci is consistent across multiple cell lines and seems independent of p53 status (i.e. wild-type or mutant p53) (Supplementary Fig. S1A). Important to note that the length of the elongated foci we observe varies across different cell lines, long elongated structures can be observed in RPE-1 cells (Fig. 1D and E), while elongated foci form, but are usually shorter in length in HCT116 or U2OS cells (Supplementary Fig. S1A and Fig. 5). This difference in length between cell lines likely reflects differences in genetic background and repair kinetics, as RPE-1 cells display slower repair kinetics, while HCT116 cells resolve the majority of RAD51 foci more rapidly (Figs 1B and 5A). In addition, RAD51 foci extend in all cell lines tested when HR is perturbed (see below).

The elongated RAD51 foci we observed were oriented away from the γH2AX-positive core of the DNA damage focus (Fig. 1E and Supplementary Fig. S1B–D), implying that the elongated RAD51 foci are associated with a broken DNA end. In addition, the lack of γH2AX-staining on the elongated structures suggests that this part of the nucleoprotein filament is free of nucleosomes, or that γH2AX has been removed. Elongated foci formed in S-phase as well as in G2 phase (Supplementary Fig. S1E). When comparing topology of the extended structures at 6 versus 16 h after irradiation, we noted that the elongated foci that remained unresolved at 16 h were longer and less spherical (Fig. 1F–H), suggesting that unresolved RAD51 foci continue to grow in length.

**Figure 2.**
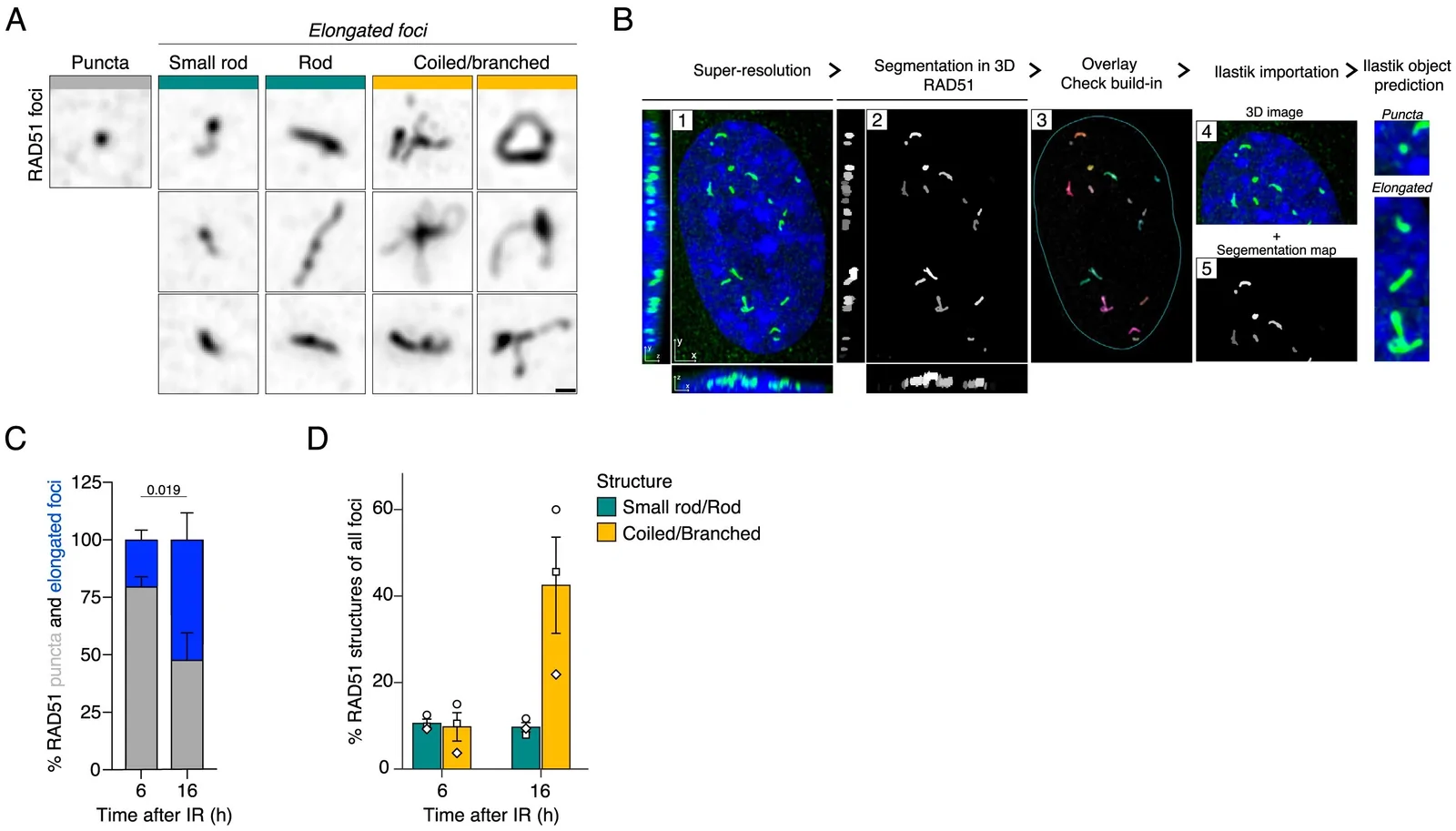
SR imaging of RAD51 reveals their intracellular localization. (**A**) Distinct topologies of RAD51 puncta/elongated foci visualized using SR microscopy. Examples of puncta (left panel), small rods (2nd row), rods (3rd row), and coiled/branches (4th and 5th rows) categories are shown (16 h, 4 Gy): scale bar, 0.5 μm. (**B**) Panels 1 and 2: machine learning-assisted segmentation of the nucleus and RAD51 structures. Lateral (left side) and orthogonal (bottom) cross sections of 3D image stacks are shown. Panel 3: maximum intensity projection of both the original SR image and the segmentation, which closely resembles the structures. Panels 4 and 5: both the original 3D SR image and segmentation map (in 3D) were imported into Ilastik. Right panels: Ilastik was trained to predict either puncta or elongated foci. (**C**) Average percentages of RAD51 puncta and elongated foci at 6 and 16 h after IR (4 Gy). Data were obtained through Ilastik (*N* = 3, *n* = >100 foci per replicate). Statistical significance of the percentage of RAD51 filaments is calculated by unpaired *t*-test using Prism (6 versus 16 h *P* = .0190). (**D**) Ilastik-based analysis of various RAD51 foci topologies in RPE-1 HALO-53BP1 cells. Small rods/rods and coiled/branches categories were made of different shapes as shown in panel (A) (6/16 h, 4 Gy) (*N* = 3, *n* = >100 foci per replicate).

Elongated RAD51 foci observed in RPE-1 cells using SR microscopy appeared in a variety of confirmations (Fig. 2A and B) closely resembling the RAD51 “filaments” observed previously by single-molecule localization microscopy in HeLa cells and yeast [18, 20]. We could discern distinct structures of elongated foci that we classified as small rods/rods, and coiled/branched structures (Fig. 2A), and we employed Ilastik machine learning to quantify these subclasses (Fig. 2B). When comparing the combined frequency of these subclasses at 6 and 16 h, we observed a clear shift in the spherical puncta/elongated foci ratio, so that roughly half of all the remaining RAD51 foci at 16 h appeared as elongated foci (Fig. 2C and Supplementary Fig. S2A). In addition, there was a significant increase in focus area (Supplementary Fig. S2C) and complexity (Fig. 2D). RAD51 length (Supplementary Fig. S2B) increased, while focus circularity decreased (Supplementary Fig. S2D) at 16 h after damage, but these differences did not reach statistical significance. The average longest path of RAD51 foci increased from ∼500 nm at 6 h to ∼750 nm at 16 h post-damage (Supplementary Fig. S2B). Using an estimated conversion of ∼2 kb ssDNA per µm [29], the length of the structures (ranging ∼500 nm to 3 µm) would correspond to ~1-6 kb of resected ssDNA. This is consistent with resection track lengths measured in human cells by END-seq, which showed average resection track lengths of ∼3.4 kb [30]. Together, this suggests that the elongated RAD51 foci we observe *in vivo* represent a physiologically relevant length of resected ssDNA.

For HR to be functional, the resected end must locate and invade a homologous template, either on the sister chromatid or, less preferentially, on the homologous chromosome. To estimate whether the elongated foci that we observe could extend beyond the sister chromatid to reach the homologous chromosome, we directly measured sister chromatid distances in RPE-1 cells using DNA-FISH in our imaging setup and find an average inter-sister distance of ~300 nm under undamaged G2 conditions. The measured distances between sister loci (Supplementary Fig. S2E) indicate that a significant subset of the elongated foci we observe exceed the expected inter-sister distance, suggesting that the longest structures may indeed extend beyond the sister chromatid and potentially engage in homology search in the broader nuclear volume. We note that at 16 h, a part of the initial DNA damage foci remain unresolved (Fig. 1B and C). The remaining RAD51 foci presumably failed to identify a suitable repair template on the nearby sister and continue to grow because of ongoing resection, potentially explaining the increase in length and complexity seen at 16 h. Possibly, these elongated foci are associated with the search for a repair template on the homologous chromosome. Taken together, these elongated RAD51 foci we observe in human cells could be relevant representations of RAD51 nucleoprotein filaments that engage in homology search beyond the sister chromatid, like the proposed role for Rad51-coated nucleoprotein filaments in yeast.

### RAD54L is required for the resolution of elongated RAD51 foci

**Figure 3.**
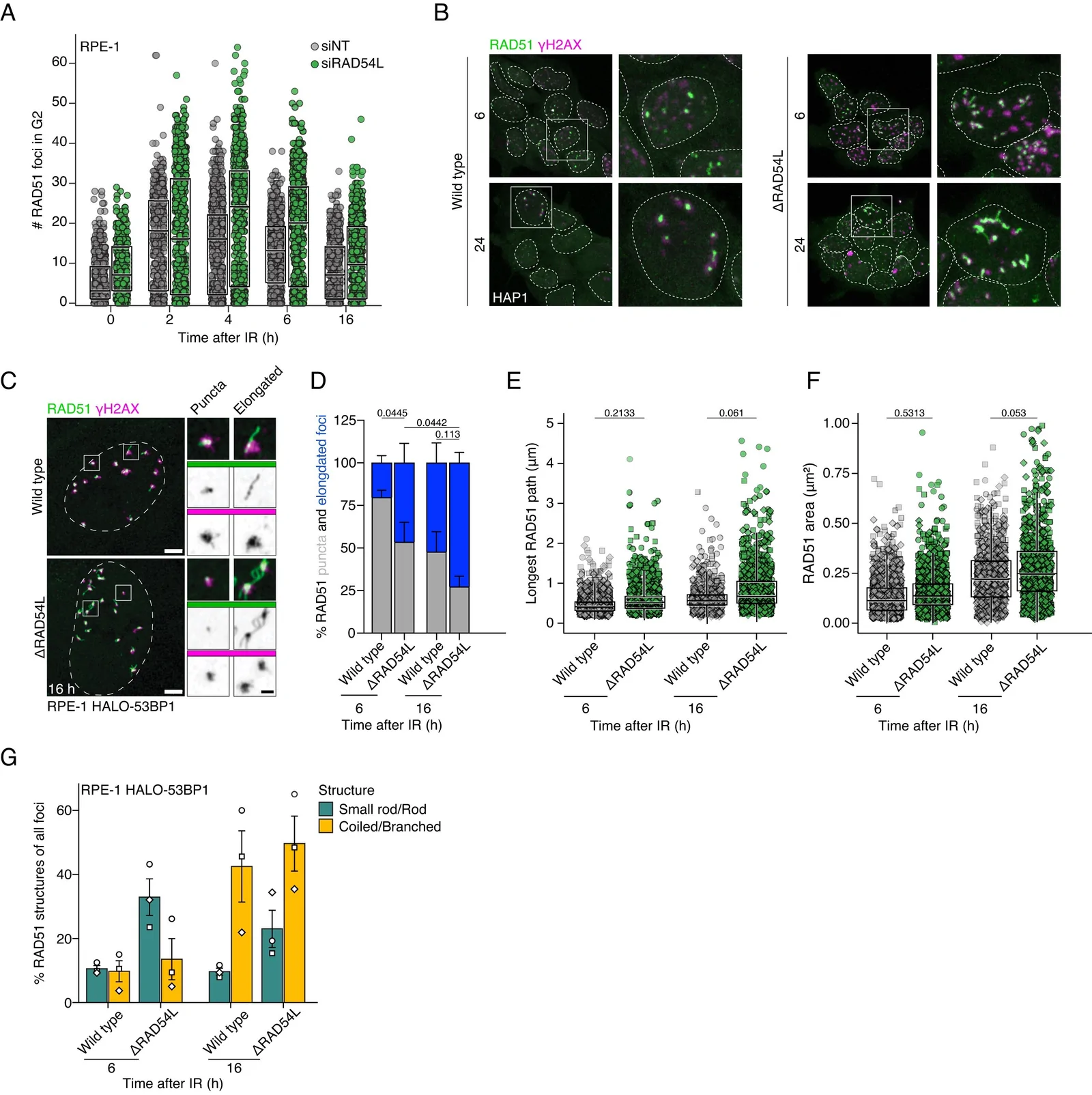
RAD54L is required for the resolution of RAD51 elongated foci. (**A**) Quantification of RAD51 foci in RPE-1 HALO-53BP1 cells treated with non-targeting (siNT) or RAD54L-targeting (siRAD54L) siRNA and imaged at different time points after irradiation (4 Gy). Foci numbers were determined in G2 phase cells. Three independent replicates are displayed. (**B**) RAD51 puncta/elongated foci and γH2AX foci in HAP1 wild-type (right four panels) and HAP1ΔRAD54L cells (left four panels) imaged by SR. Cells were irradiated with 2 Gy and fixed 6 (top four panels) or 24 h later (lower four panels). (**C**) γH2AX foci and RAD51 puncta/elongated foci observed by SR microscopy at 16 h after IR (4 Gy) in wild-type and ΔRAD54L RPE-1 HALO-53BP1 cells. Scale bars, 4 μm. Scale bars insets, 1 μm. (**D**) Average percentages of RAD51 elongated foci in wild-type and ΔRAD54L RPE-1 HALO-53BP1 cells at 6 and 16 h after irradiation (4 Gy). Data were obtained using Ilastik (*N* = 3). Wild-type percentages at 6 and 16 h depict the same data as in Fig. 2C. WT versus ΔRAD54L at 6 h: *P* = .0445; WT versus ΔRAD54L at 16 h: *P* = .113; ΔRAD54L 6 h versus ΔRAD54L 16 h: *P* = .0442. (**E**) Ilastik analysis of the longest RAD51 path (μm) of individual RAD51 foci in wild-type and ΔRAD54L RPE-1 HALO-53BP1 cells at 6 and16 h after irradiation (4 Gy). Each dot represents an individual RAD51 focus. Data are presented with individual measurements depicted with a different symbol per replicate. Box plots representing center line, median; box limits, 25th and 75th percentiles. WT versus ΔRAD54L at 6 h: *P* = .2133; WT versus ΔRAD54L at 16 h: *P* = .061. (**F**) Ilastik-based analysis of the area (μm^2^) of all RAD51 foci in wild-type and ΔRAD54L RPE-1 HALO-53BP1 cells at 6 and 16 h after irradiation (4 Gy). Each dot represents a single RAD51 focus. Each dot represents an individual RAD51 focus. Data are presented with individual measurements depicted with a different symbol per replicate. Box plots: center line, median; box limits, 25th and 75th percentiles. WT versus ΔRAD54L at 6 h: *P* = .5313; WT versus ΔRAD54L at 16 h: *P* = .053. (**G**) Average percentages of different RAD51 foci topologies at 6 and 16 h after irradiation (4 Gy) in wild-type and ΔRAD54L RPE-1 HALO-53BP1 cells. Wild-type assemblies at 6 and 16 h depict the same data as Fig. 2D. Small rods/rods and coiled/branched were used as categories. Data were obtained using Ilastik. (D–F) *N* = 3, *n* = >700 foci examined per replicate, distinct symbols indicate the replicates. Statistical significance was assessed by two-way ANOVA with Šídák”s multiple comparisons test.

Several co-factors have been shown to control the formation, stabilization, and strand invasion of RAD51-nucleoprotein filaments. RAD54L is a member of the SWI2/SNF2 family of DNA-dependent ATPases that acts at several stages during the homology search. It was shown to stabilize the RAD51 nucleoprotein filament, to be required for D-loop formation and D-loop dissociation through its branch migration activity, and to remove RAD51 from heteroduplex DNA after strand invasion [31–34]. Early after DNA damage induction, the amount of RAD51-positive foci was not affected by loss of RAD54L (Fig. 3A, *t* = 2 h), indicating that recruitment of RAD51 to DNA breaks is not affected by loss of RAD54L. However, while RAD51 foci number started to drop at 4 h after damage induction in the controls (siNT), we noted a further rise in RAD51 foci in the RAD54L-depleted cells (Fig. 3A). This implies that the first breaks that engage in RAD51-dependent repair are repaired within 4 h after break induction. There was a further reduction in RAD51 foci visible in control cells at 6-16 h after damage induction (Fig. 3A), suggesting that the time it takes to complete RAD51-dependent repair varies significantly across different DNA breaks, possibly as a consequence of variable amounts of time required to locate the proper repair template. Importantly, depletion of RAD54L from RPE-1 cells resulted in a clear delay of RAD51-dependent repair, as evidenced by an increase in residual RAD51 foci at the later time points (Fig. 3A), with the most notable differences visible at the 4- and the 6-h time-points.

Given that deletion of RAD54L can inhibit the resolution of RAD51-foci, we wanted to investigate whether this affects the formation and/or resolution of the elongated foci. To test this, we generated RAD54L knockout clones in HAP1 (HAP1∆RAD54L) and RPE-1 background (RPE-1∆RAD54L) (Supplementary Fig. S3A and B). Consistent with the repair defect we observed after depletion of RAD54L by siRNA in RPE-1 cells (Fig. 3A), HAP1 cells in which RAD54L was knocked out displayed an increased sensitivity to irradiation (Supplementary Fig. S3C). We observed a striking difference in the number and size of elongated RAD51 foci at 6 and 24 h after damage induction, when comparing the HAP1 and HAP1∆RAD54L cell lines (Fig. 3B). While we could observe very few elongated foci at 6 h after irradiation in the parental HAP1 cell line, many of the RAD51 foci displayed an elongated structure in the RAD54L knockout cells (Fig. 3B). Importantly, this increase in foci elongation is not due to increased cell death in the RAD54L knockout cells, as cell viability was measured and found to be comparable between HAP1 wild-type and RAD54L knockout cells at 6 and 16 h after 2 Gy irradiation (Supplementary Fig. S3D). A similar increase in RAD51 foci elongation was observed in the RPE-1∆RAD54L cell line (Fig. 3C and D, and Supplementary Fig. S3F). Also, absence of RAD54L resulted in less circular, longer foci that covered more area, both at the 6 and the 16 h time points (Fig. 3E and F, and Supplementary Fig. S3E). Thus, when RAD51 foci elongation is allowed, but RAD51-dependent repair is perturbed by the depletion of RAD54L, elongated foci accumulate at earlier time points and can become longer and more complex (Fig. 3G). This indicates that RAD51 foci grow in length over time and adopt more complex topological structures (coiled/branched) (Fig. 2A and D). This process is further enhanced when strand invasion or D-loop dissociation is inhibited by the loss of RAD54L (Fig. 3G). This suggests that the elongated structures could represent pre-synaptic RAD51 nucleoprotein filaments probing the nucleoplasm or represent post-synaptic RAD51 nucleoprotein filaments that fail to be resolved.

**Figure 4.**
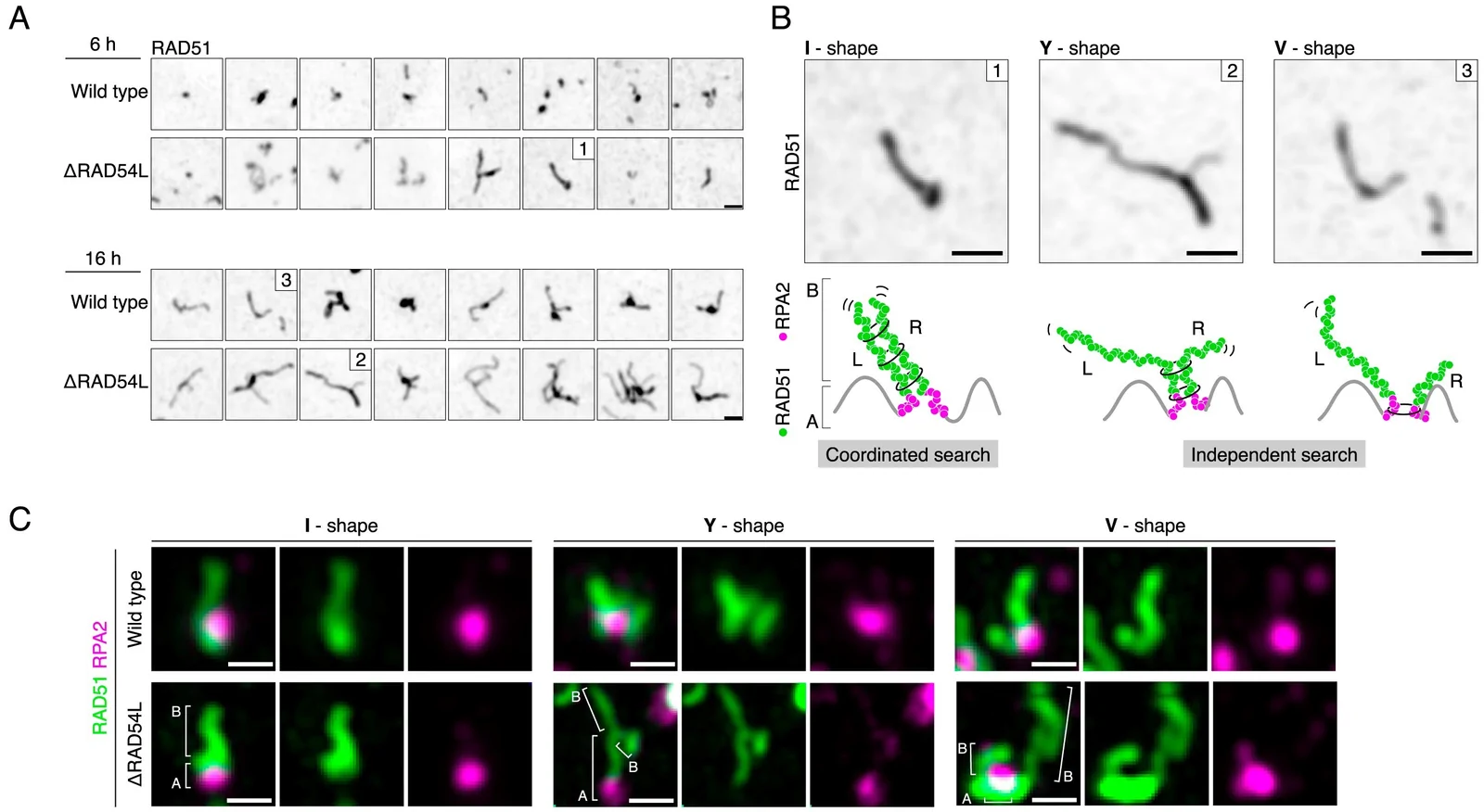
Visual evidence of independent versus coordinated search modes. (**A**) Individual RAD51 puncta/elongated foci in wild-type (top panels) and ΔRAD54L RPE-1 HALO-53BP1 cells (bottom panels) exposed to irradiation (4 Gy) for the indicated times, imaged by SR microscopy. Scale bars, 1 μm. (**B**) Magnifications of the three images from panel (A) depicting examples of (1) I-shaped, (2) Y-shaped, or (3) V-shaped RAD51 elongated foci. Scale bars, 0.5 μm. Underneath the respective images two models for homology search. The broken DNA seeks for its donor via coordinated [panel (1)] or/and independent [panels (2) and (3)] homology search mechanisms. (**C**) RAD51 and RPA2-foci in wild-type (top panels) and ΔRAD54L RPE-1 HALO-53BP1 cells (lower panels) imaged by SR microscopy at 16 h after IR (4 Gy). Scale bar, 0.5 μm.

Interestingly, we frequently observed elongated foci adopting Y- or V-shape configurations (Fig. 4A and B, and Supplementary Fig. S3G). Possibly, these Y/V-shapes could represent independent homology search events initiated by two resected DNA ends flanking the break, in contrast to the more commonly observed I-shapes that could represent the coordinated search of both ends (Fig. 4A and B). Using an antibody against γH2AX, a well-known marker of DNA breaks, we found that γH2AX staining is commonly observed at the base of the Y/V-shapes (Supplementary Fig. S3G). RPA2 likewise decorates this DNA damage core in an ordered, circular arrangement in both wild type as well as ∆RAD54L cells (Fig. 4C). Thus, while these data can certainly not be taken as proof for coordinated versus independent homology search in living cells, they do suggest that direct visualization of RAD51 in real-time in living cells could possibly allow one to study these different modes of homology search. Taken together, our data indicate that while RAD51 foci initially form as puncta, and can extend into elongated foci over time, particularly when strand invasion is perturbed. These elongated foci grow in length over time and extend away from the core of a DNA damage focus, potentially reflecting a visual representation of the homology search process when a suitable template is not found within the area covered by the initial RAD51 puncta.

### Cohesin, not CTCF, modulates RAD51-mediated repair

**Figure 5.**
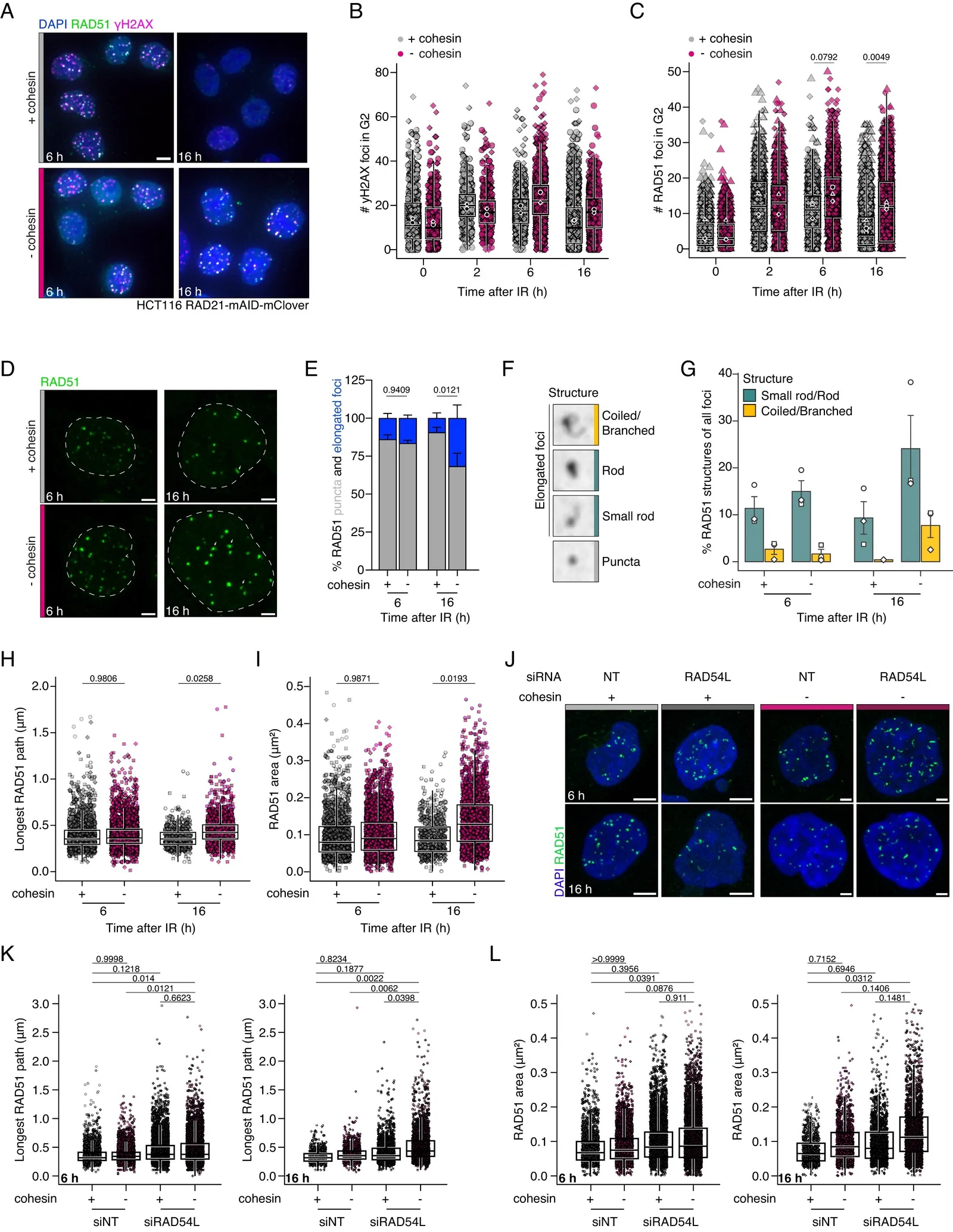
Cohesin, not CTCF, modulates RAD51-mediated repair. (**A**) γH2AX foci and RAD51 puncta/elongated foci observed by wide-field microscopy in HCT116 RAD21-mAID-mClover cells exposed to irradiation (4 Gy, 6 and 16 h). Scale bars, 5 μm. Cells were either treated with DMSO (+cohesin, top panels) or 5-Ph-IAA to deplete RAD21 (−cohesin, bottom panels) 1 h before irradiation. Quantification of the amount of γH2AX- (**B**) or RAD51-foci (**C**) at the indicated time points after irradiation (4 Gy) in cells treated with DMSO (+cohesin) or 5-Ph-IAA to deplete RAD21 (−cohesin) 1 h before irradiation. Each dot represents an individual cell, distinct symbols (circle, diamond triangle) are used for each replicate. Box plots: center line, median; box limits, 25th and 75th percentiles (*t* = 0, 2 *N* = 2, *t* = 6, 16 h *N* = 3), *n* = >100 cells per replicate. (C) Statistical analysis is performed using unpaired *t*-test in PRISM. +cohesin versus −cohesin at 6 h: *P* = .0792; +cohesin versus −cohesin at 16 h: *P* = .0049. (**D**) RAD51 foci in HCT116 RAD21-mAID-mClover cells with (+cohesin, top panels) or without cohesin (−cohesin, lower panels) exposed to irradiation [4 Gy, 6 (left panels) and 16 h (right panels)] imaged by SR microscopy. Scale bars, 2 μm. (**E**) Average percentages of RAD51 puncta/elongated foci at 6 and 16 h after irradiation (4 Gy). Data were obtained using Ilastik (*N* = 3, at least *n* = >100 foci per replicate). +cohesin versus −cohesin at 6 h: *P* = .9409; +cohesin versus −cohesin at 16 h: *P* = .0121. (**F**) Insets of images with examples of the four categories of RAD51 foci topologies observed in HCT116 RAD21-mAID-mClover cells. Puncta, Small rods and rods, and coiled/branched were used as categories. (**G**) Average percentages of rods and coiled/branched assemblies at 6 and 16 h after irradiation (4 Gy). RAD51 assemblies were scored as shown in panel (F). Data were obtained using Ilastik. (**H**) Ilastik-based analysis of the longest RAD51 path of individual RAD51 foci in cells treated as in panel (D). Each dot represents a single RAD51 focus. Data are presented with individual measurements and box plots. Centre line, median; box limits, 25th and 75th percentiles. +cohesin versus −cohesin at 6 h: *P* = .9806; +cohesin versus −cohesin at 16 h: *P* = .0258. (**I**) Ilastik-based analysis of the area (μm^2^) of all RAD51 foci in cells treated as in panel (D). (H, I) Each dot represents a single RAD51 focus. Data are presented with individual measurements and box plots. Centre line, median; box limits, 25th and 75th percentiles. +cohesin versus −cohesin at 6 h: *P* = .9871 +cohesin versus −cohesin at 16 h: *P* = .0193. (E, H, I) Statistical significance was assessed by two-way ANOVA with Šídák’s multiple comparisons test (*N* = 3, *n* = >400 foci examined per replicate, distinct symbols indicate the replicates). (**J**) SR images of RAD51 foci in HCT116 RAD21-mAIDmClover cells exposed to irradiation (4 Gy, 6 and 16 h). Cells were treated for 48 h with siNT or siRAD54L followed by 5-Ph-IAA to deplete RAD21 (−cohesin, left panels) or DMSO (+cohesin, right panels) addition 1 h before irradiation (4 Gy, 16 h). Scale bars, 2 μm. (**K**) Ilastik-based analysis of the longest RAD51 path (μm) of individual RAD51 foci in cells treated as in panel (J). Each dot represents a single RAD51 focus. Centre line, median; box limits, 25th and 75th percentiles. (**L**) Ilastik-based analysis of the area of all RAD51 foci in cells treated as in panel (J). Each dot represents a single RAD51 focus. Data are presented with individual measurements and box plots. Centre line, median; box limits, 25th and 75th centiles. For panels (K) and (L) statistical significance was assessed by two-way ANOVA with Turkey’s multiple comparisons test (*N* = 3, *n* = >400 foci examined per replicate, distinct symbols indicate the replicates).

Our data thus far suggest that the elongated RAD51 foci we observe might arise when a suitable homologous template cannot be found nearby and pairing needs to be established between distant sequences. Cohesin facilitates physical association and alignment of sister chromatids from replication onward and loss of cohesin subunits has been shown to impair HR in various organisms [35–45]. To test whether cohesin controls the behavior of RAD51 foci, we used an HCT116 cell line that expresses the essential cohesin subunit RAD21 endogenously tagged with a degron [46]. This allows for rapid degradation of RAD21 upon the addition of 5-Ph-IAA (Supplementary Fig. S4A). To test repair kinetics after cohesin depletion, 5-Ph-IAA was added 1 h before irradiation to allow for complete RAD21 degradation, rendering the cohesin complex non-functional, as evidenced by a clear mitotic cohesin-defect (Supplementary Fig. S4B). Therefore, we refer to the RAD21 depletion as cohesin depletion. Cohesin deficiency did not affect the global induction of damage as measured by the initial appearance of γH2AX (Fig. 5A and B), but the resolution of γH2AX foci at later stages was impaired (Fig. 5A and B). Cohesin loss led to a prominent impairment of the resolution of RAD51 foci (Fig. 5A and C). While a significant fraction of the RAD51 foci appears to resolve between 6 and 16 h in HCT116 cells expressing cohesin, this resolution was blocked by the depletion of cohesin (Fig. 5A–D). This indicates that the repair defect induced by cohesin loss is mediated via a defect in RAD51-mediated HR, as shown before in HeLa cells after X-ray-induced DNA damage in G2 [41]. Next, we examined the effects of cohesin loss on RAD51 foci topology using SR imaging. At 6 h post-damage, average RAD51 focus size was indistinguishable from control conditions, but at 16 h, we more frequently observed elongated structures in cells that lacked cohesin, alongside longer RAD51 structures that covered more area and are slightly less spherical (Fig. 5D–I and Supplementary Fig. S4E). This indicates that cohesin loss influences RAD51 dynamics and foci resolution in human cells, implying that cohesin loss should impair DNA damage repair in S/G2. Consistent with this notion, we find that loss of cohesin has a major effect on mitotic entry after DNA damage (Supplementary Fig. S4C and D). Elongated foci in HCT116 cells tend to be shorter than those observed in RPE-1 cells (compare Fig. 5H and Supplementary Fig. S2B), which we attribute to differences in genetic background, proliferation rates, and/or repair kinetics. Upon depletion of cohesin, we observed an increase in complex topologies at 16 h after irradiation, suggesting that cohesion normally constrains foci architecture (Fig. 5D–I). These data demonstrate that loss of cohesin impairs RAD51-dependent homology search and perturbs mitotic progression under damaged conditions.

In addition to linking sister chromatids, cohesin also forms DNA loops on single chromatids, a role which has also been suggested to contribute to DNA repair by defining topologically associated domains (TADs) [47–49], which are flanked by CTCF sites. TADs restrict the spreading of γH2AX and aid in DNA repair [43, 44, 47, 50, 51]. To test whether the repair defects observed after cohesin loss are mediated by CTCF, we depleted CTCF at 1 h before irradiation in HCT116 CTCF-mAIDClover cells [52] and observed efficient CTCF depletion (Supplementary Fig. S4G). While depletion of cohesin significantly affected RAD51 foci resolution, CTCF depletion had no impact on RAD51 foci resolution (Supplementary Fig. S4H) and also did not affect RAD51 foci length at 16 h post-damage (Supplementary Fig. S4I). Also, after low-dose irradiation (2 Gy) no difference in mitotic entry was observed when comparing CTCF-depleted HCT116 cells with unperturbed controls (Supplementary Fig. S4J). Overall, these findings indicate that the essential role of cohesin in HR is independent of CTCF-anchored DNA looping.

### Cohesin and RAD54L are required for distinct steps of the homology search

To investigate whether RAD54L and cohesin function at the same step during homology search, we performed siRNA-mediated knockdown of RAD54L in HCT116-RAD21-mAID-Clover cells. SR imaging showed that in RAD54L/RAD21 co-depleted HCT116 cells, elongated RAD51 foci were even more pronounced than after cohesin or RAD54L depletion alone (Fig. 5J–L). Particularly at 16 h after damage, co-depletion of cohesin and RAD54L decreased circularity (Supplementary Fig. S4K), and enhanced foci length and area (Fig. 5K and L). This implies that homology sampling by the RAD51 nucleoprotein filament is impaired at multiple independent levels in the absence of RAD54L and cohesin. Thus, RAD54L/cohesin activities are needed for homology search, and the two have additive activities in this pathway.

### Elongated RAD51 foci recruit MND1

To obtain more insights into the real-time dynamics of RAD51 foci in human cells, we tried to produce a fluorescently tagged variant of RAD51. However, all fluorescently tagged versions of RAD51 that we produced interfered with normal repair. We therefore took an alternative approach, based on our recent observations that MND1 acts as a cofactor in RAD51-mediated repair in human somatic cells and that its dynamic localization can be traced in living human cells using GFP-MND1 [22]. To validate the use of GFP-MND1 as a proxy for RAD51 activity, we further characterized the functionality of the construct. We could show that expression of GFP-MND1 in MND1 knockouts restored expression of HOP2 (Supplementary Fig. S5A) and restored the cellular capacity to resolve DNA damage-induced 53BP1 and RAD51 foci (Supplementary Fig. S5B and C). Also, expression of GFP-MND1 reverted sensitivity to neocarzinostatin of MND1 knockouts and reverted their mitotic entry defect after irradiation (Supplementary Fig. S5D and E). Taken together, these results indicate that the GFP-MND1 construct is fully functional and suitable as a live-cell proxy for RAD51-mediated HR activity.

**Figure 6.**
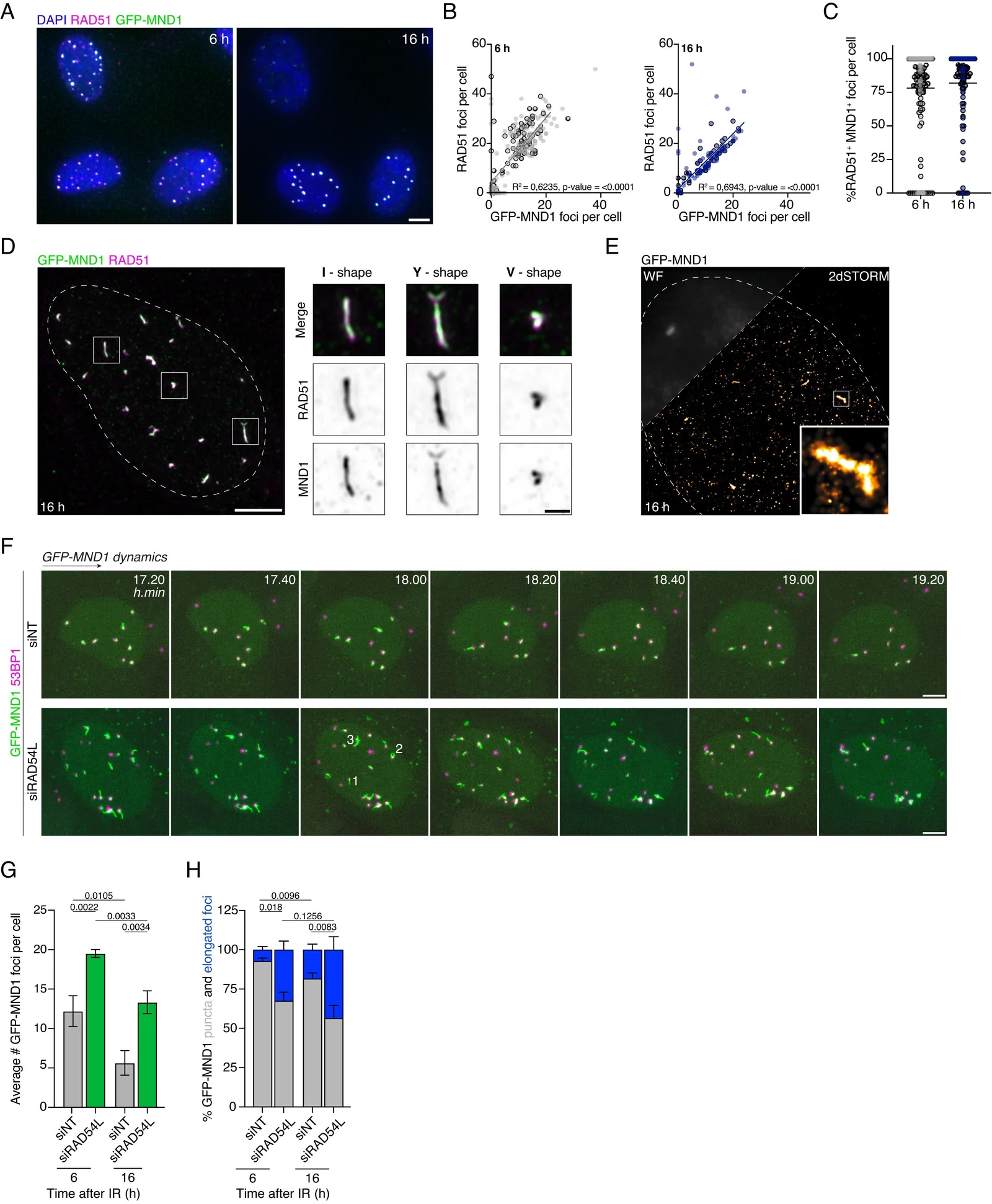
RAD51 elongated foci recruit MND1. (**A**) Examples of wide-field images of wild-type RPE-1 HALO-53BP1 GFP-MND1 cells exposed to irradiation (4 Gy) and fixed at 6 (left panel) or 16 h (right panel) after irradiation. Cells were immunostained with anti-RAD51, anti-GFP, and DAPI to stain DNA. Scale bars, 5 μm. (**B**) Number of RAD51 foci versus GFP-MND1 foci detected at 6 h (left plot) and 16 h (right plot) after 4 Gy IR in the same cell. Slope and *P*-value of the correlation are shown in the graph. (**C**) Quantification of the percentages RAD51^+^ foci that are also MND1^+^ at 6 (gray dots) and 16 h (blue dots) post damage (*N* = 2, *n* = >50 cells per replicate). (**D**) GFP-MND1- (green) and RAD51-foci (magenta) in RPE-1 HALO-53BP1 GFP-MND1 cells showing co-localization. Scale bars, 5 μm. Magnifications of examples of I-shaped, Y-shaped, and V-shaped RAD51 (magenta)/MND1 (green) elongated foci. Insets: scale bar, 1 μm. (**E**) Wide-field (WF) and 2DSTORM image of the same wild-type RPE-1 HALO-53BP1 GFP-MND1 cell immunostained with GFP to visualize a GFP-MND1-positive elongated focus at 16 h post 4 Gy. (**F**) Stills from a live-cell imaging experiment of RPE-1 HALO-53BP1 RPA1-mScarlet (not shown) GFP-MND1 cells exposed to irradiation (4 Gy) for the indicated times. Cells were treated for 48 h with a siNT (top panels) or siRAD54L (lower panels) before irradiation. Scale bars, 5 μm. (**G**) Average percentages of MND1 foci at 6 and 16 h post damage (*N* = 3). (**H**) Average percentages of MND1 elongated foci at 6 and16 h post damage (*N* = 3). Statistical significance [panels (G) and (H)] was assessed by two-way ANOVA with Šídák’s multiple comparisons test.

Indeed, when examining RAD51 foci observed at 6 and 16 h after irradiation, we find that GFP-MND1 colocalizes with RAD51 in damage-induced puncta, also in cells lacking RAD54L (Fig. 6A and Supplementary Fig. S6A and B). We find that the numbers of foci converge over time, and that the fraction of double-positive structures rises above 80% at 16 h post-damage (Fig. 6B and C). SR microscopy revealed that GFP-MND1 is also present on the elongated RAD51 foci (Fig. 6D). The GFP-MND1 foci display similar variety in topological structures as we observed for RAD51 foci (Supplementary Fig. S6A and B). In addition, GFP-MND1 is also present on the elongated RAD51 foci that can be observed in I, Y, or V-shaped structures (Fig. 6D), and that grow longer and become less spherical at later time points after irradiation (Supplementary Fig. S6C), similar to what we observed for the elongated RAD51 foci. 2D-STORM imaging of RPE-1 cells expressing GFP-MND1 revealed a clear elongated appearance of MND1-positive foci at 16 h post-damage (Fig. 6E). Elongated GFP-MND1 foci form on the core of a 53BP1 focus (Supplementary Fig. S6D). Imaging in fixed cells shows that this core stains positive for γH2AX (Supplementary Fig. S6E and F) and BRCA1/BRCA2 (Supplementary Fig. S6G). Taken together, these data demonstrate that GFP-MND1 consistently co-localizes to the RAD51 foci during formation and elongation and could potentially be used to track the dynamics of these foci in living cells.

To investigate whether elongated foci formation is a peculiar feature of GFP-tagged MND1, we obtained an RPE-1 cell line expressing exogenous MND1 with a HALO-tag (Supplementary Fig. S7A). This tagging strategy has the added benefit that it is compatible with the FUCCI system [53] (Supplementary Fig. S7A–D) allowing for real-time cell cycle stratification during foci formation. Using RPE-1-FUCCI-HALO-MND1 cells we could show that elongated HALO-MND1 foci are formed in S and G2 phase cells, but not in G1 (Supplementary Fig. S7B and D), very similar to what we have seen for RAD51.

Next, we asked whether the GFP-MND1 foci respond to the depletion of RAD54L. We conducted live-cell imaging with and without RAD45L siRNA and like what we observed for RAD51-positive foci in RAD54L-depleted cells, GFP-MND1 foci grew larger (Fig. 6F and H). Moreover, the resolution of MND1-positive foci appears to be inhibited (Fig. 6F and G) and the relative percentage of MND1-coated elongated foci was enhanced after depletion of RAD54L (Fig. 6H). Similarly, resolution of HALO-MND1 foci was also impaired upon loss of cohesin (Supplementary Fig. S7E–G), all consistent with what we observed for RAD51. Together, these findings suggest that both RAD54L and cohesin are critical for the timely resolution of RAD51/MND1-coated foci, and their depletion results in enhanced RAD51/MND1 foci elongation.

### Real-time dynamics of MND1 foci

**Figure 7.**
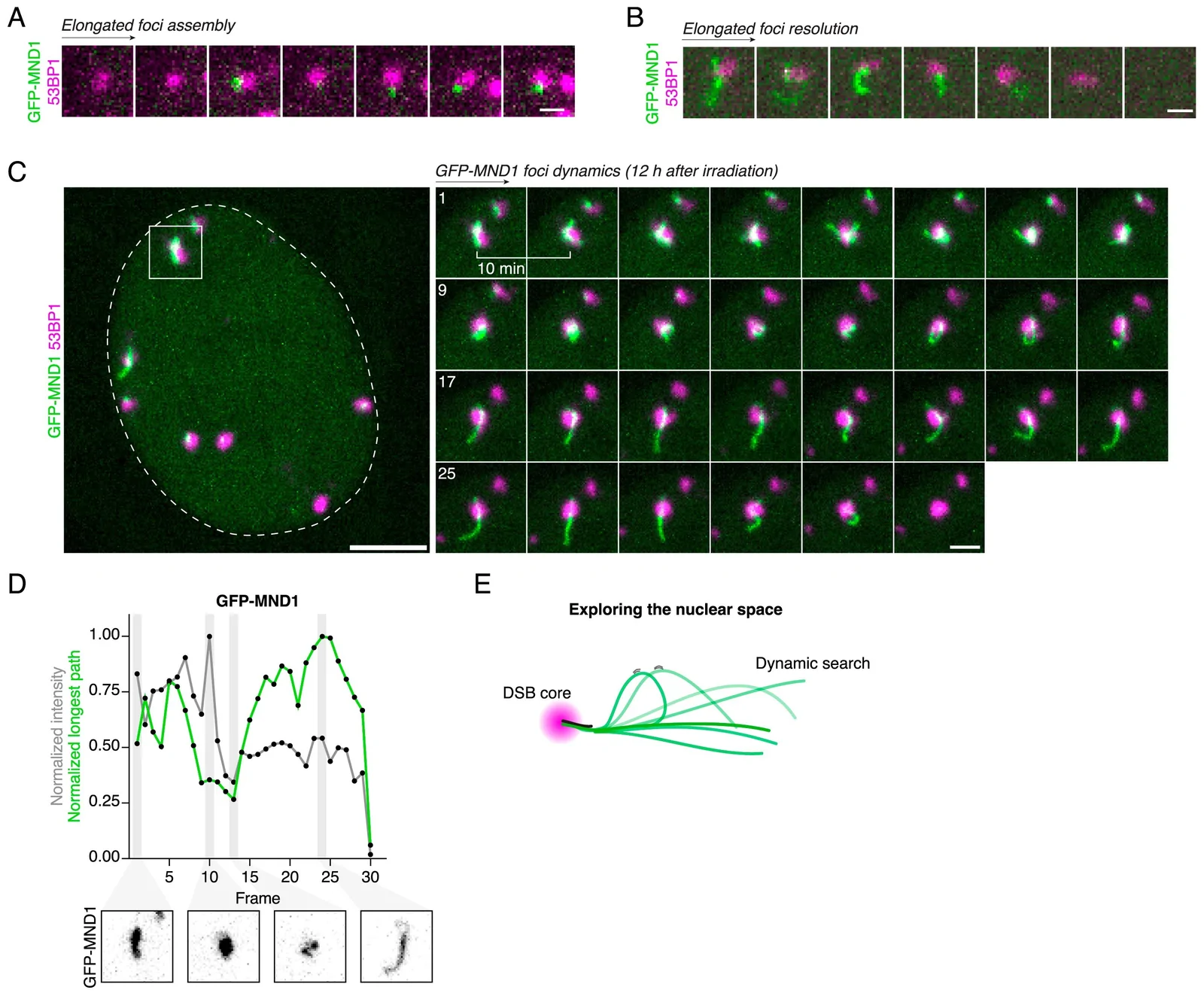
Real-time dynamics of MND1 foci. Stills from a live-cell imaging experiment showing the assembly (**A**) or resolution (**B**) of GFP-MND1-positive elongated foci on a 53BP1 focus in a wild-type RPE-1 HALO-53BP1 RPA1-mScarlet (not shown) GFP-MND1 cell exposed to irradiation (4 Gy) for the indicated time. Time interval between images is 10 min. Scale bars, 0.5 μm. (**C**) Stills from a live-cell imaging experiment showing the assembly, dynamic movement, and resolution of an MND1-GFP-positive elongated focus from and into an 53BP1 focus in a wild-type RPE-1 HALO-53BP1 RPA1-mScarlet (not shown) GFP-MND1 cell exposed to irradiation (4 Gy) for the indicated time. The time interval between images is 10 min. Scale bars, 5 μm and insets, 2 μm (Supplementary Video S1). (**D**) Normalized fluorescence intensity and normalized longest path of the GFP-MND1 assembly over time of the images shown in panel (C). Below insets of start of the elongated foci (frame = 1), highest intensity (frame = 10), lowest intensity (frame = 13), and longest GFP-MND1 path (frame = 24) (Supplementary Video S2). (**E**) Model: schematic representation of RAD51 elongated foci (green) that could form from the “core” of the DSB (magenta) and dynamically explore the nuclear environment, navigating through the crowded 3D nucleus to seek its homologous sequence.

To gather further evidence that the elongated RAD51/MND1-positive foci represent homology search in action in living human cells, we monitored GFP-MND1 behavior after irradiation over longer periods of time using live-cell imaging. To track the core of the break in these experiments, we made use of a HALO-tagged version of 53BP1. To this end, we expressed GFP-MND1 in a RPE-1 cell line containing endogenously tagged HALO-53BP1. At 5–30 min after ionizing radiation (4 Gy), single repair foci appeared, marked by loading of HALO-53BP1, consistent with previous reports [54, 55]. Time-lapse imaging showed that 53BP1-positive foci are initially devoid of GFP-MND1, but recruit GFP-MND1 to a focal site somewhere on the periphery of the 53BP1 focus (Fig. 7A). These foci elongate (Fig. 7A–C), some of which eventually resolve, and can be followed by resolution of the 53BP1 focus (Fig. 7B). GFP-MND1 foci appear to extend away from the core and display a constant process of stretching and retracting over time (Fig. 7C and D) (Supplementary Video S1), potentially to locate the donor template. Live-cell imaging showed that these elongated foci can span very large distances and once formed, GFP-MND1 foci are highly dynamic, undergoing continuous changes in orientation, bending, and shape, transitioning between rod-like and more complex configurations over time (Fig. 7B). Quantification of GFP-MND1 intensity over time showed that long periods of relatively constant fluorescence intensity alternate with short periods in which intensity sharply drops/increases (Fig. 7). This suggests that periods in which an elongated focus explores nuclear space alternate with periods in which the elongated focus (diss)assembles. As shown in Fig. 7D, a transient collapse of an elongated focus into a bright circular focus (Fig. 7D frame 10) is followed by a rapid drop in focus intensity, after which the focus gains intensity and extends (Fig. 7D, compare frame 10 and 24). These observations suggest that GFP-MND1 dynamics reflect active structural remodeling as all classes of shapes could be observed within a single time-lapse (Supplementary Video S2). Detailed analysis of foci dynamics revealed two distinct behavioral classes. First, short-lived elongated foci that are present for only one or two frames, indicating that transient foci extension and retraction events could occur on a timescale of <10 min. Second, long-lived elongated foci that persist over longer periods and appear to explore the nuclear space over long distances (Supplementary Video S3). Given the time interval between frames in our experiments, it is not unlikely that we still underestimate three-dimensional movements.

Together, these observations suggest that RAD51/MND1 foci display a spectrum of dynamic behaviors, ranging from transient exploratory extensions to more stable structures, which may reflect different stages or outcomes of the homology search process. This, combined with movements in different directions away from the core of the focus could allow the nucleoprotein filaments to explore very large volumes in the nucleus (Fig. 7E). This highly dynamic nature of MND1 mirrors the characteristics of Rad51 “filaments” observed in yeast [20] and represents the first live visualization of homology search in mammalian cells. Importantly, we observed multiple instances where the elongated MND1-positive focus resolved, followed by the resolution of the original 53BP1 focus. This indicates that the elongated foci we observe can be used in a successful repair event that displaces 53BP1, implying that the highly dynamic MND1-positive structures observed represent nucleoprotein filaments engaged in long-range homology search.

**Figure 8.**
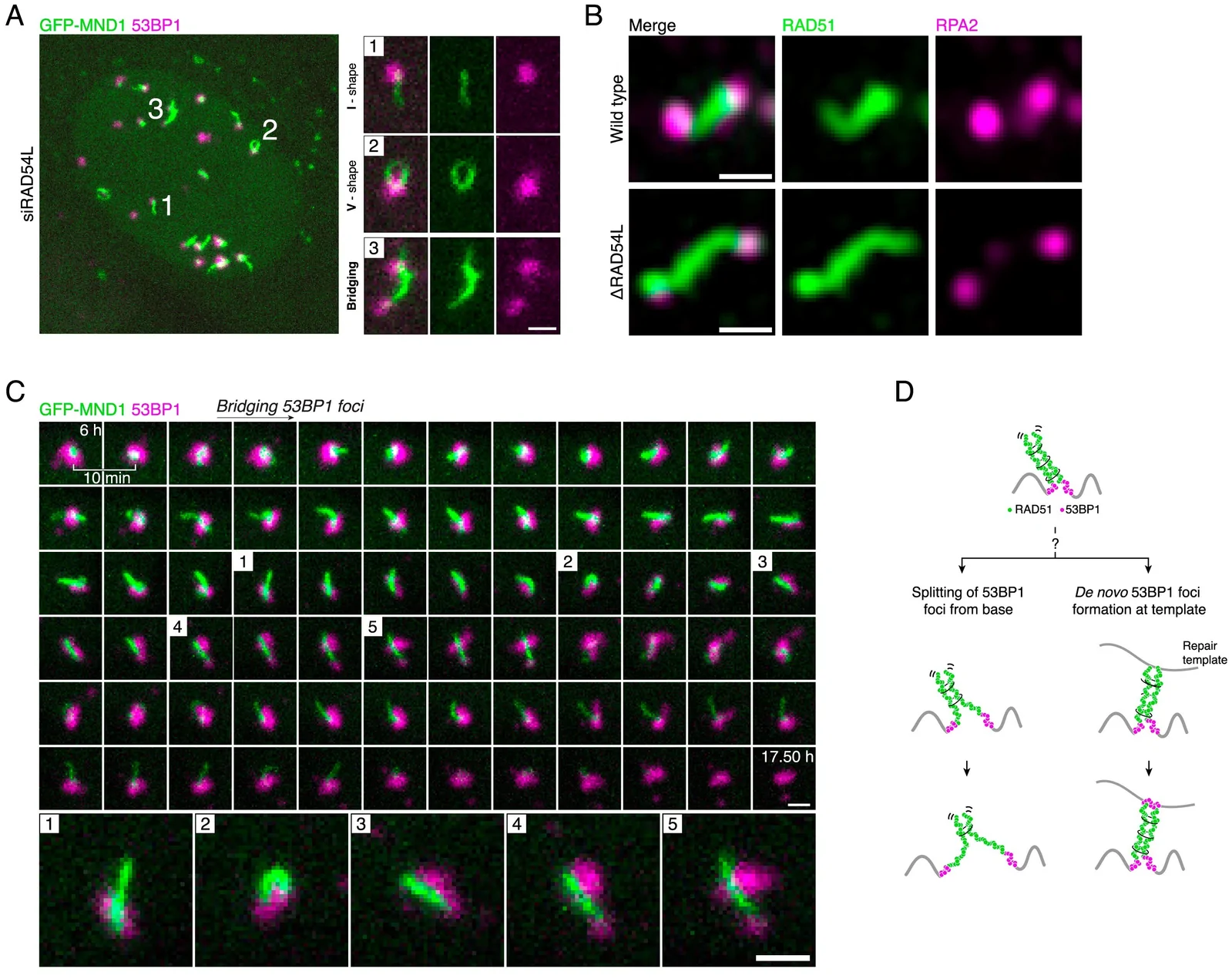
Real-time dynamics of MND1 foci. (**A**) Magnifications (small panels) from a still (large panel) from a live-cell imaging experiment depicting examples of (1) I-shape, (2) V-shape, or (3) bridging GFP-MND1 elongated foci with 53BP1 foci. Scale bar, 1 μm. (**B**) SR of immunostained RAD51 and RPA2 in irradiated (4 Gy, 16 h) wild-type and ΔRAD54L RPE-1 HALO-53BP1 cells highlighting the bridging event of the elongated foci. Scale bar, 0.5 μm. (**C**) Stills from a live-cell imaging experiment following a wild-type RPE-1 HALO-53BP1 RPA1-mScarlet (not shown) GFP-MND1 filament exposed to irradiation (4 Gy) for the indicated times (10 min time interval). Scale bars, 0.5 μm. (**D**) Two potential bridging modes of RAD51 elongated foci connecting two 53BP1 foci. (Left) Existing 53BP1 foci split from a parental focus at the DSB core, generating two foci. (Right) 53BP1 foci form *de novo* at the template, independent of pre-existing foci. Both pathways would result in 53BP1-bound foci associated with the elongated foci.

During these live-cell imaging experiments, we could observe formation of I-shaped, as well as V-shaped GFP-MND1-positive structures that grew out from the 53BP1 core (Fig. 8A). We also occasionally observed GFP-MND1-positive structures that appear to bridge multiple 53BP1-positive foci (Fig. 8A). Analysis in fixed cells showed that bridging structures can also be observed connecting two RPA2-positive (Fig. 8B) or γH2AX-positive dots (Supplementary Fig. S8A). To investigate these bridging events in more detail, we performed time-lapse imaging in RPE-1 cells expressing HALO-53BP1, GFP-MND1, and RPA1-mScarlet, the latter protein to mark ssDNA. Using these cells, we could show that both ends of the GFP-positive elongated focus that connect two 53BP1 foci are also positive for RPA1-mScarlet (Supplementary Fig. S8B and C) suggesting there is ssDNA on either side of these bridging structures. Important to note that while there invariably was a RPA2 focus associated with one end of the elongated focus, the other connected RPA2 focus could also be observed along the length of the elongated MND1 focus, rather than at the end (Supplementary Fig. S8D and E). This implies that these elongated foci have retained patches of RPA that have not (yet) been replaced by RAD51, or that RPA is recruited to the ssDNA in the D-loop that forms when the RAD51/MND1 nucleoprotein filament invades the dsDNA template (Supplementary Fig. S8F).

When following connecting structures in time lapse imaging experiments, we could observe examples where an originally “one-sided” elongated focus gave rise to a second, less prominent 53BP1 focus that was brought in proximity to the original focus as the elongated focus retracted (Fig. 8C), suggestive of a search and capture event in HR. Upon careful evaluation of these connecting events, we note two distinct phenotypes. First, some connecting dots appear smaller and lower in intensity than the original damage site, suggesting they may represent a secondary structure rather than an independent damage focus (Fig. 8C, Supplementary Fig. S8C and G, and Supplementary Video S4). Second, we observe events suggestive of the splitting of a single 53BP1 focus into two, where the intensity of the original focus appears redistributed between the two resulting dots (Supplementary Fig. S8H and Supplementary Video S5). We can only speculate what causes the second 53BP1 focus to appear, the nucleoprotein filament potentially establishes an interaction with another 53BP1 focus that was not in the same focal plane, tethered ends might become (partially) untethered thus leading to separation of the 53BP1 signal at the base of the resected ends (Fig. 8D). Alternatively, D-loop formation might lead to 53BP1 recruitment at the site of strand invasion (Fig. 8D).

## Discussion

In this study, we use quantitative high-content SR microscopy to visualize DNA repair proteins active in HR. Using live-cell imaging, we uncover a previously unreported phenomenon in living human cells: the formation of dynamic elongated foci at DNA break sites. We can visualize the formation of elongated RAD51 foci in fixed and elongated MND1 foci in live cells. We propose that these elongated foci represent an intermediate state of HR of DNA breaks that search for homologous DNA sequences. Such a search mechanism has recently also been observed in yeast [20], suggesting a shared principle for the search for donor templates.

We show that elongated RAD51/MND1 foci originate from a confined DNA damage core, stained by γH2AX, RPA1, RPA2, and 53BP1. These elongated foci arise after induction of DNA damage and become prominent at 6 h post-damage. We show that loss of RAD54L or loss of cohesin, two factors that previously have been identified to control HR repair, results in more pronounced foci elongation. RAD54L, a member of the SWI2/SNF2 family of DNA-dependent ATPases, has important pre- and postsynaptic functions. It stabilizes RAD51 nucleoprotein filaments, facilitates D-loop formation and dissociation through its branch migration activity, and promotes the dissociation of RAD51 from the D-loop or heterologous sequences [31–34]. Cohesin by contrast builds loops to promote the local search for homology [43, 44], facilitates the formation of γH2AX domains [43, 44, 47, 50], and confers sister chromatid cohesion [56, 57]. We reveal that loss of either of these factors results in extended foci elongation compared to wild-type cells. Future studies are required to test which function(s) of RAD54L and cohesin do restrict foci elongation, and whether the elongation occurs at a pre- or postsynaptic stage.

The data that we obtained after depletion of RAD54L indicate that foci elongated further when strand invasion or D-loop dissociation is perturbed. This may imply that many of the HR events that take place in the first 6 h in the cells used in this study occur at a relatively short range, possibly within the original puncta. This is best illustrated by our observation that prominent foci elongation is observed at 6 h in RAD54L-depleted cells, while their number is still very low in parental RPE-1. This observation implies that a subset of the RAD51-positive foci can resolve at short range in parental RPE-1 and therefore do not form elongated foci that search beyond the boundaries of the puncta.

We used live-cell imaging of GFP-MND1 to address the movement of the elongated foci with respect to the DNA damage core. We observe that GFP-MND1 foci indeed originate and extend from this 53BP1-positive core, that the elongated foci adopt various conformations, and that the elongated focus eventually retracts toward this core. The length of the elongated foci allows them to span the distance between sister chromatids observed previously [7]. Although the sister chromatid is most commonly used as a repair template, HR can sometimes also result in translocation to the homologous chromosome. Together, these findings support a model in which elongated foci represent HR intermediates that search for homology.

We observe that RAD51/MND1 foci can achieve lengths at 16 h after damage that supersede the distance to the sister chromatid, possibly allowing the broken end to reach the repair template on the homologous chromosome as well. It is possible that the gradual extension of elongated foci represents the shift from local search on a proximal sister chromatid, toward long-range/global search for a sister chromatid displaced by DNA looping or the homologous chromosome. Indeed, recent experiments in yeast reveal that the search of a DNA break for donor sequences is restricted to its local environment shortly after break induction [58]. An alternative explanation for the progressive extension of RAD51/MND1 foci is that breaks engage in HR, but that the subsequent search for a donor template sequence fails. Excessive resection of these unrepairable breaks would then result in long stretches of ssDNA that are coated with RAD51/MND1. Elongated RAD51/MND1 foci therefore may thus also represent products of failed (local) repair. However, as these elongated foci are also seen to resolve, presumably representing the repair of the break, we favor the model in which at least a subset of these elongated foci represent HR intermediates that aid in the successful localization of repair donor templates. Our findings suggest that the loss of cohesin results in longer and more complex RAD51 structures, which may reflect a shift in homology search template from the sister chromatid toward the homologous chromosome. This is consistent with the proposed dual role of cohesin in maintaining chromosome integrity, where cohesin can promote DSB repair between sister chromatids but it can also actively suppress damage-induced recombination between homologs [59]. In the absence of cohesin, this suppressive function is lost, potentially allowing the elongated focus to extend further into the nuclear volume in search of the homologous chromosome as an alternative repair template. Our data cannot distinguish between the contributions of loop-extruding and cohesive cohesin to the observed phenotypes. However, Teloni *et al*. recently demonstrated that these represent two functionally distinct mechanisms: loop-cohesin defines a broad megabase-scale sampling in direct vicinity of the DSB, while cohesive cohesin concentrates locally at the break to direct RAD51 filaments toward the sister chromatid [43]. We show that loss of CTCF neither affect resolution of RAD51 foci, nor does it result in increased foci length, indicating that the role of looping-cohesin in resolution of RAD51 foci is not exerted via binding to CTCF.

It is likely that excessive resection of long stretches of ssDNA allows for the extension of elongated RAD51 foci. However, a focus could also extend when MND1 and/or RAD51 oligomerize independent of ssDNA binding [60], when the filament unfolds from a folded conformation into a more linearly-stretched structure [61], or when aligned RAD51 bundles (double-coated RAD51 filaments) shift toward lateral filaments (single-coated RAD51 filament) [16]. This latter scenario could also explain the thicker central regions observed in the branched GFP-MND1 structures. It therefore will be interesting to test which of these activities control focus elongation.

Notably, the elongated foci often adopt I-, V-, or Y-shaped conformations during the homology search process. Although much more characterization is needed, the I-shape may represent two paired broken ends moving together, while V- and Y-shaped structures could represent the independent movement of two complete or partial loose ends, respectively. Single-strand Hi-C studies in yeast [58] suggests that the two ends of a break often perform search for homologous sequences in a coordinated manner, but that they indeed not always remain together. Also, our live-cell imaging data reveal two distinct scenarios associated with 53BP1 connecting events that warrant further consideration. In the first phenotype, a secondary 53BP1 dot appears, which is smaller and lower in intensity than the core damage site. We speculate that this could arise either through an interaction of the extending elongated focus with a 53BP1 focus residing in a different focal plane, or through *de novo* 53BP1 recruitment driven by D-loop formation at the site of strand invasion, which may locally remodel chromatin and trigger a damage response. In the second scenario, a single 53BP1 focus appears to split into two dots with redistributed intensity, which we hypothesize may reflect the physical separation of the two DNA break ends at the ssDNA/dsDNA junction, with RAD51/MND1 coating the resected ssDNA, while the broken ends remain tethered to one another. Both phenomena are intriguing and suggest that the bridging behavior of RAD51/MND1 foci is more complex than initially appreciated, warranting dedicated investigation in future studies.

In addition, we sometimes observe that elongated RAD51 foci are flanked by RPA on both ends of the elongated structure. We speculate that this conformation reflects the capture event during homology search; one end of the elongated structure representing the original puncta where RPA binds to ssDNA in the single-stranded overhang, while the other end could represent RPA binding to ssDNA in the D-loop of the synaptic complex. This would be consistent with the described role for RPA in stabilizing the DNA pairing established by RAD51 [62]. In this model, RPA would then stabilize the ssDNA when homology is found by the RAD51 filament to prevent the formation of secondary structures [63, 64].

Here, we developed advanced four-color live-cell imaging of fluorescently tagged with GFP-MND1 as an HR marker, allowing for the first time to visualize homology search in live cells. The ability to track the dynamics of HR in living cells provides valuable opportunities to screen for genetic factors and small molecules that influence the homology search process. Through this approach, we uncovered a role for RAD54L and cohesion in regulating focus elongation. The transient and reversible elongated RAD51/MND1 foci visualized here for the first time in human cells serve as a powerful tool for understanding the dynamics of homology search and the impact of perturbations on genome stability in health and disease.

## Supplementary Material

gkag863_Supplemental_Files

## Data Availability

Analysis scripts and object classifiers used (https://doi.org/10.5281/zenodo.16621349). ImageJ software (NIH) (https://imagej.net/ij/download/). TIDE (Tracking of Indels by Decomposition) was used to estimate the editing efficiency of the crRNAs (http://shinyapps.datacurators.nl/tide/). RAD51 structures were measured with Ilastik (v.1.3.2). Any additional information required to reanalyze the data reported in this paper is available from the lead contact upon request.

## Notes

Present address: Princess Máxima Center for Pediatric Oncology, Heidelberglaan 25, Utrecht 3584 CS, The Netherlands

Present address: Miltenyi Biotec, Leiden, The Netherlands, Dellaertweg 9C, Leiden 2316 WZ, The Netherlands
