# Supplementary material for: The dynamics of RAD51 foci formation and elongation in living human cells
Source: Nucleic Acids Res. 2026 Sep 11;54(17):gkag863. doi: 10.1093/nar/gkag863 (PMC13563089; doi:10.1093/nar/gkag863)
Supplement: Supplementary file 1 [file gkag863_supplemental_files.zip › Suppl.Figures_1-8.pdf]

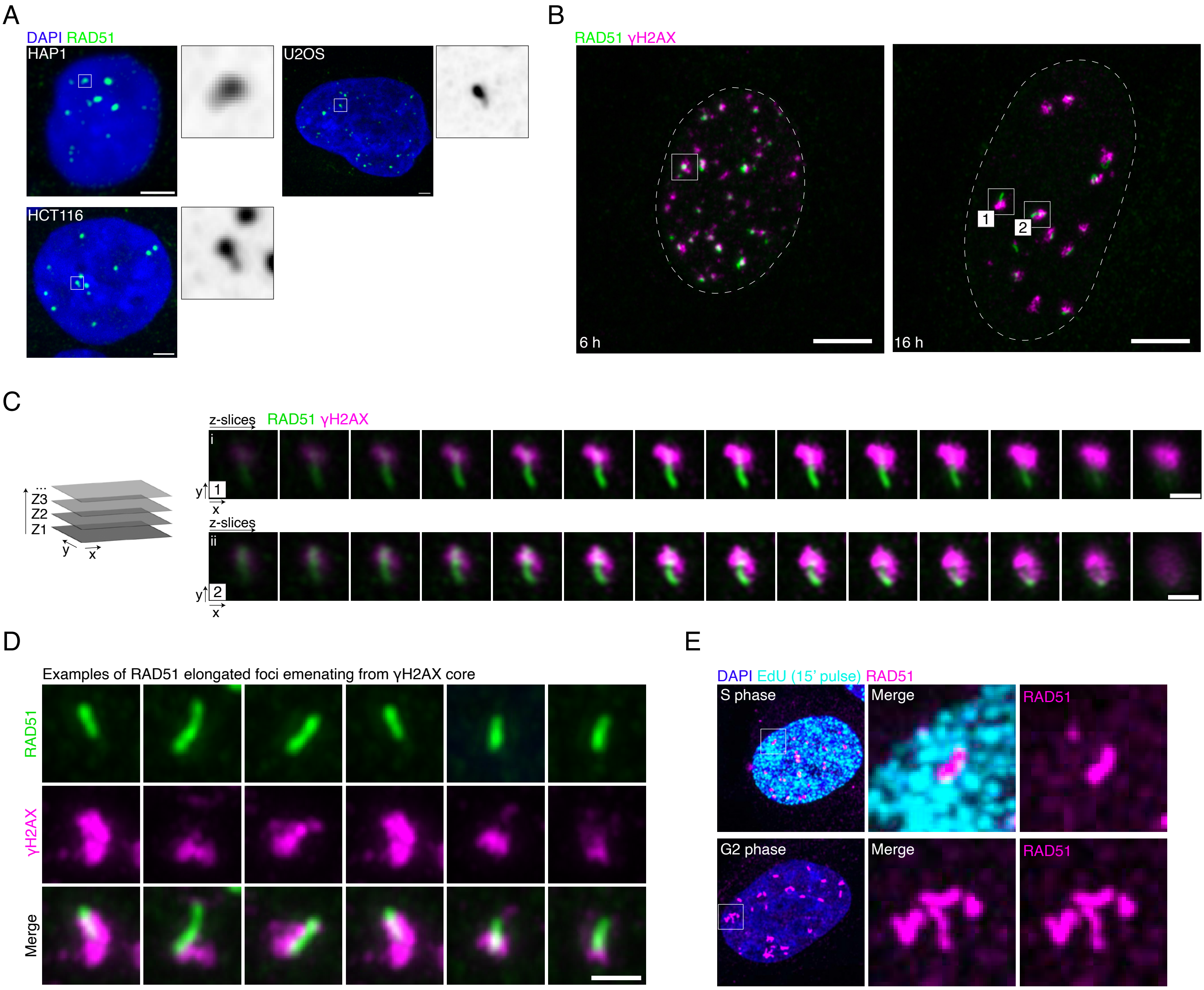

Supplemental figure 1. RAD51 forms elongated foci at sites of DNA damage.
Friskes et al.

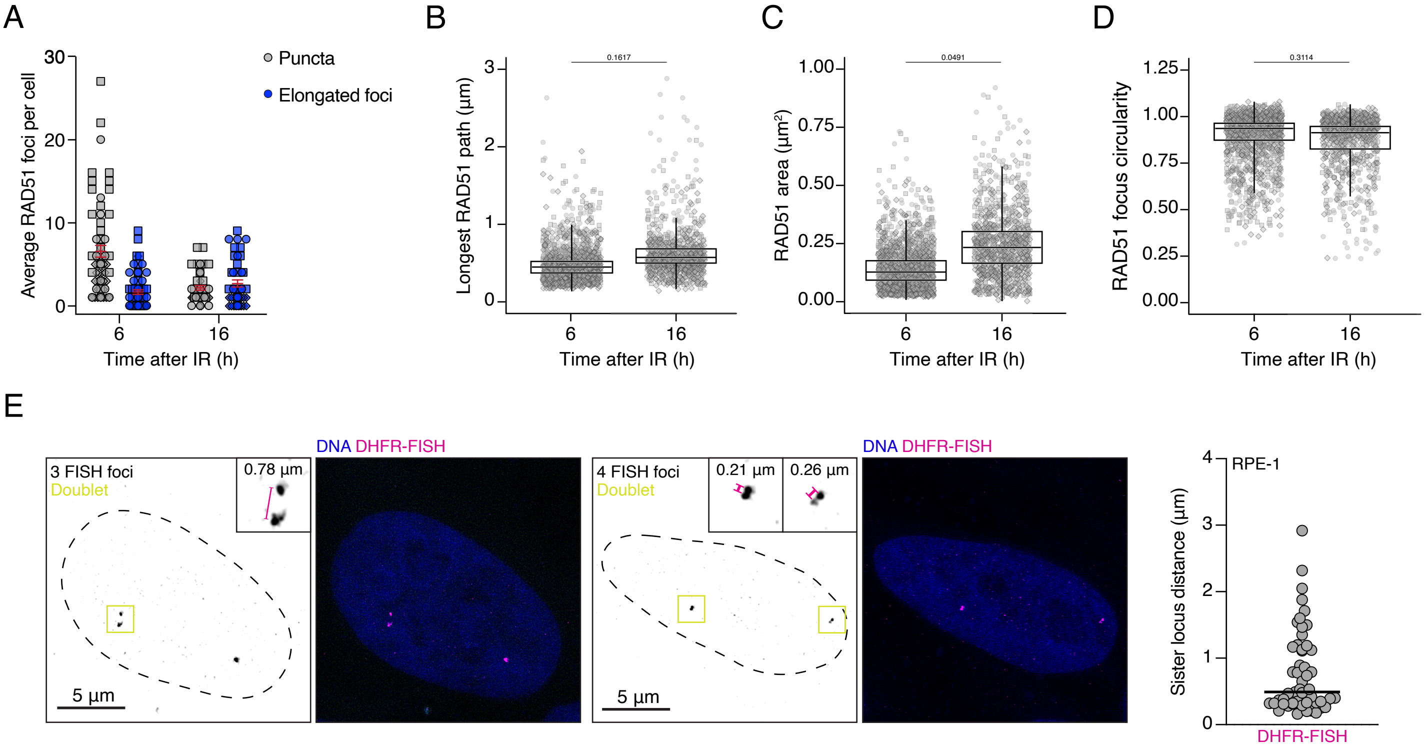

Supplemental figure 2. RAD51 forms elongated foci at sites of DNA damage.
Friskes et al.

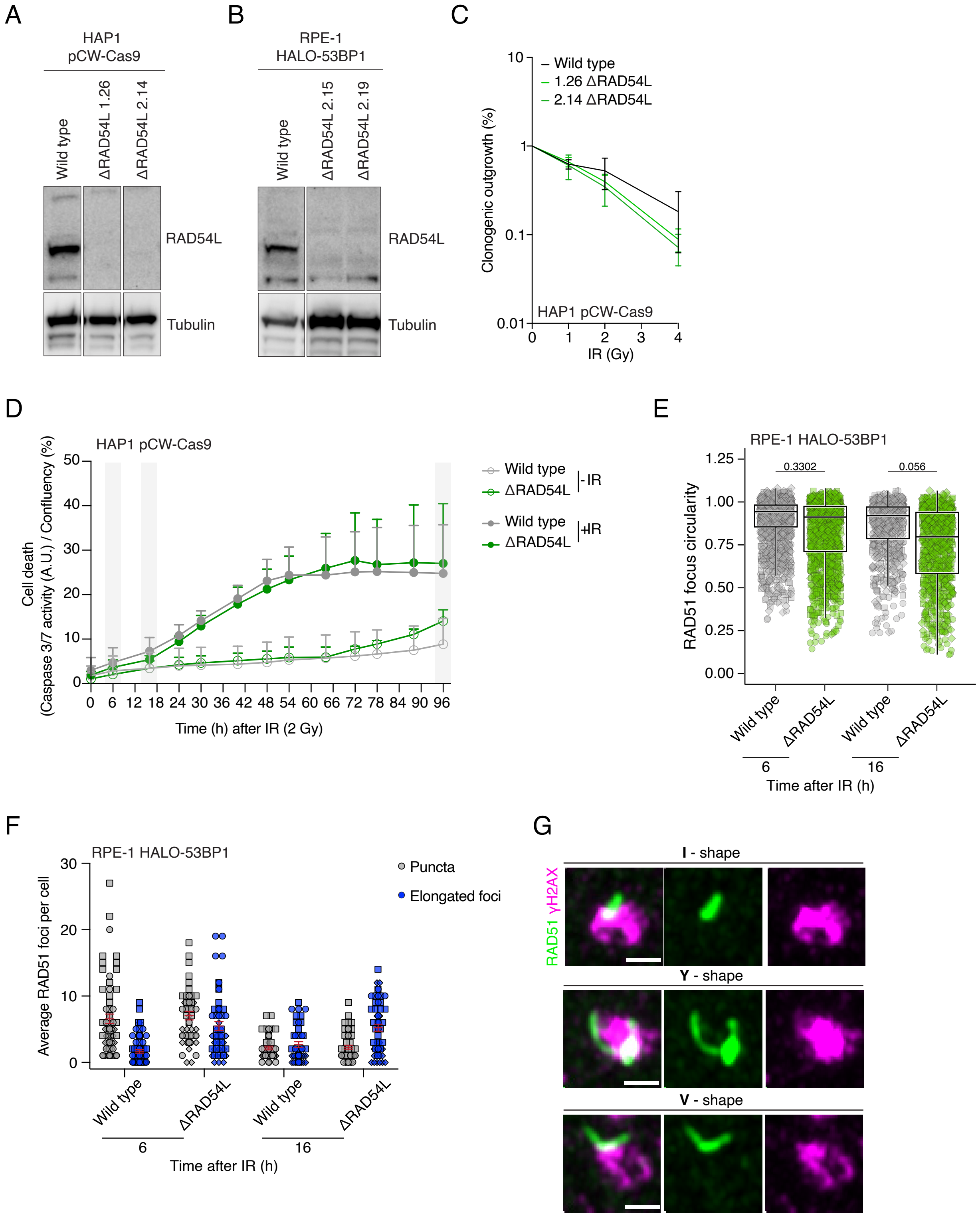

Supplemental figure 3. RAD54L is required for the resolution of RAD51 elongated foci.
Friskes et al.

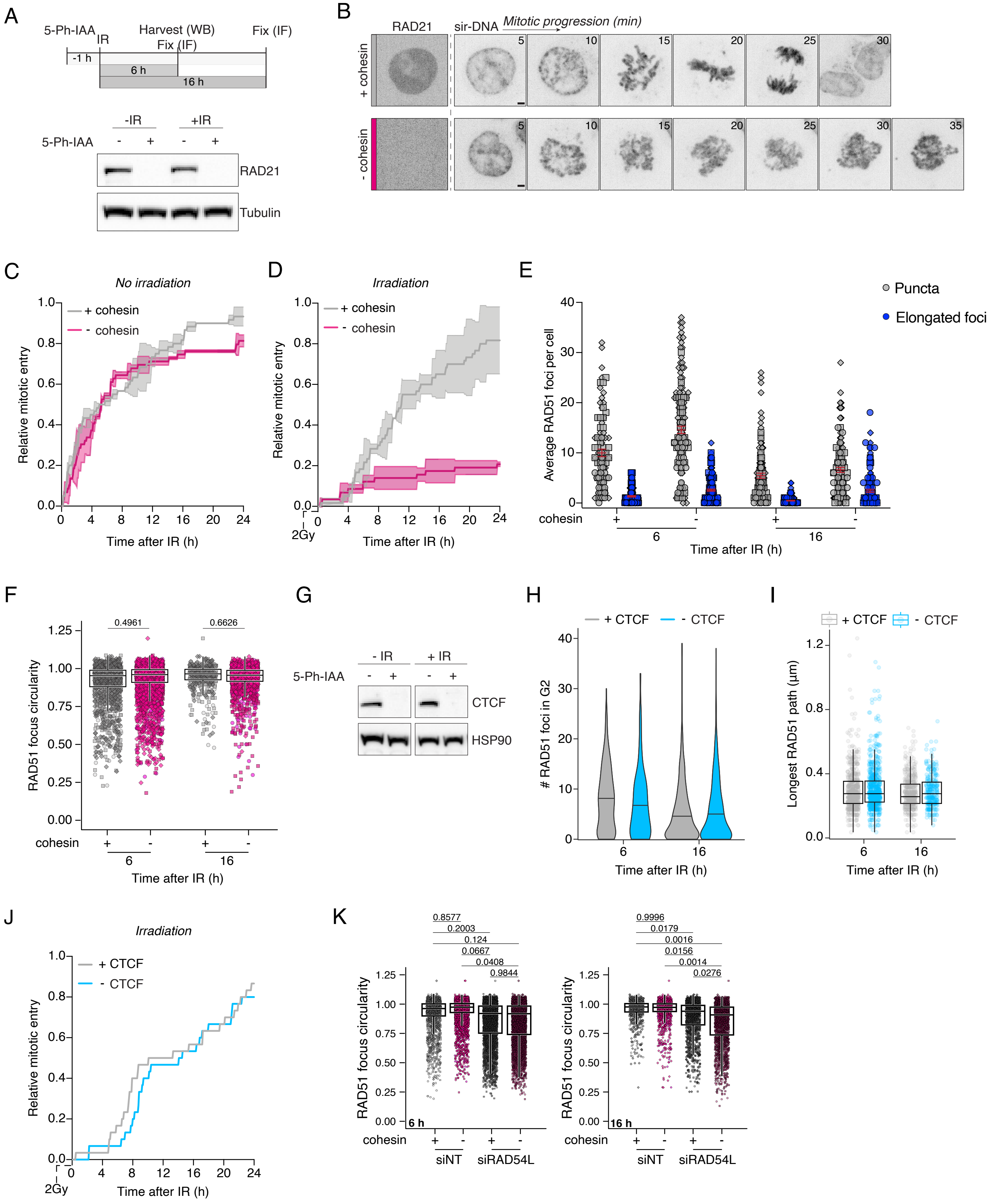

Supplemental figure 4. Cohesin, not CTCF, controls RAD51-mediated repair.
Friskes et al.

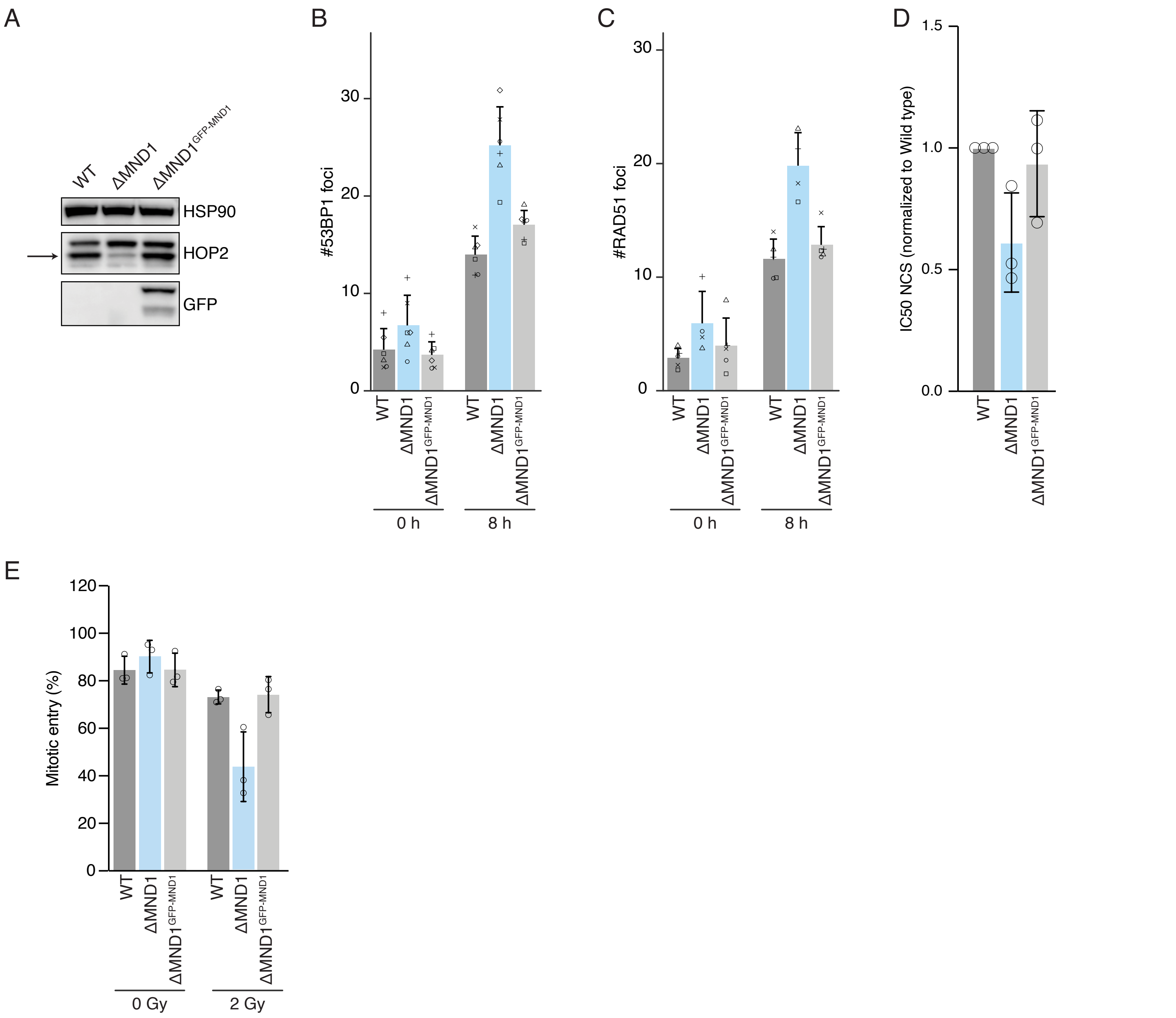

Supplemental figure 5. GFP-MND1 rescues the MND1 knock-out phenotype and can serve as a proxy for RAD51. Friskes et al.

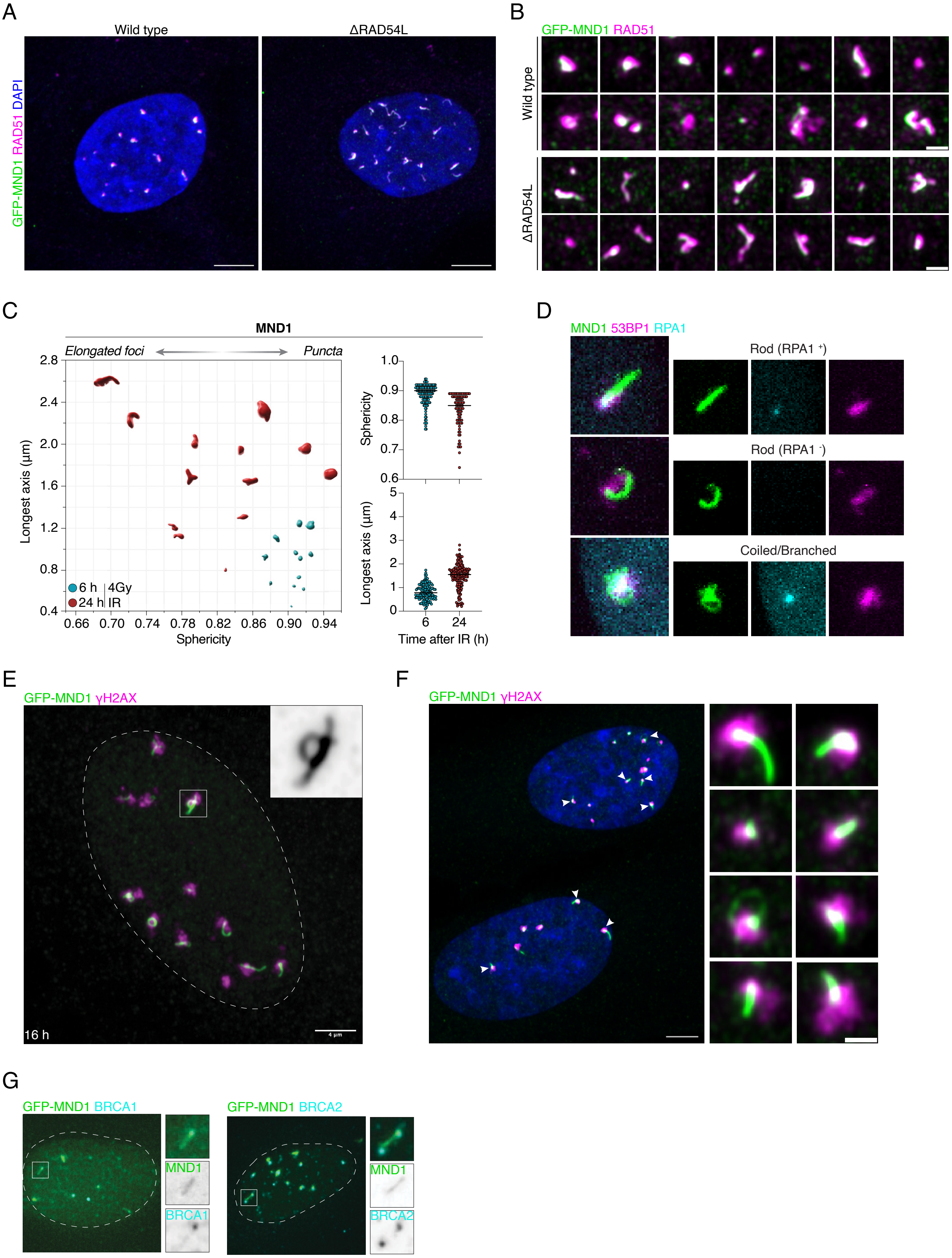

Supplemental figure 6. RAD51 elongated foci recruit MND1.
Friskes et al.

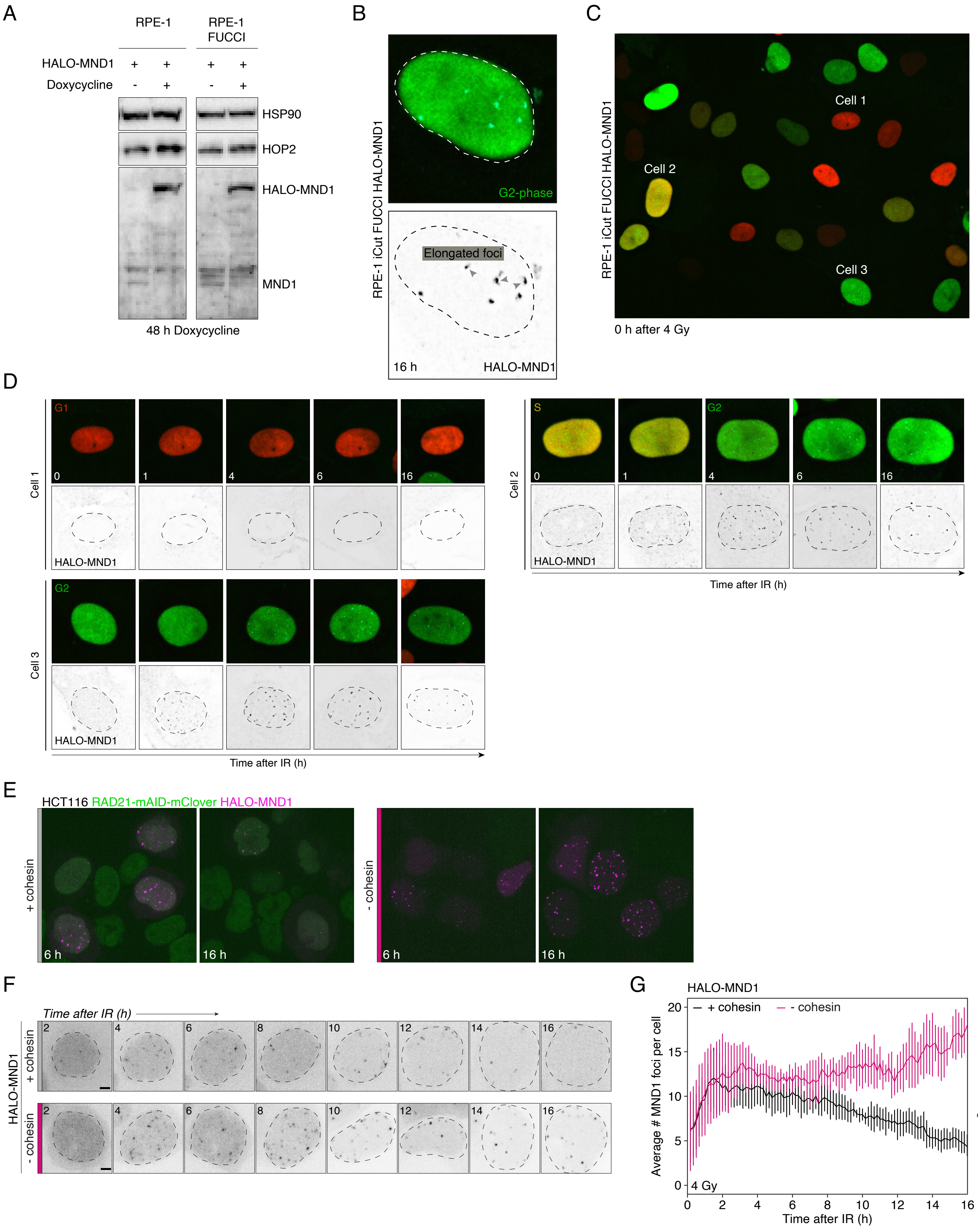

Supplemental figure 7. Elongated HALO-MND1 foci in S and G2 phase.
Friskes et al.

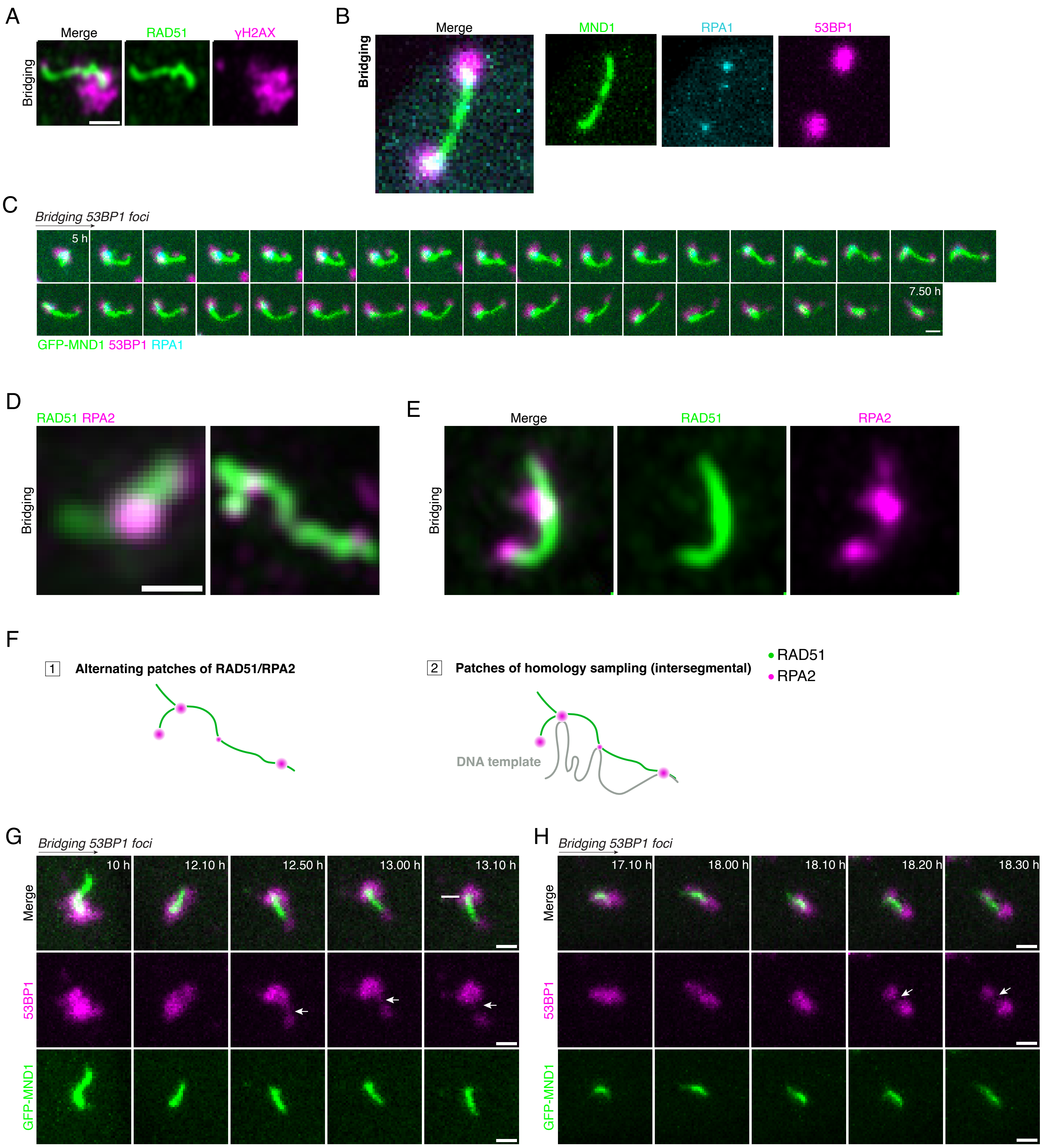

Supplemental figure 8. Bridging events in homology search.
Friskes et al.
