# Supplementary material for: The dynamics of RAD51 foci formation and elongation in living human cells
Source: Nucleic Acids Res. 2026 Sep 11;54(17):gkag863. doi: 10.1093/nar/gkag863 (PMC13563089; doi:10.1093/nar/gkag863)
Supplement: Supplementary file 1 [file gkag863_supplemental_files.zip › Supplemental figure legends[85].docx]

**SUPPLEMENTAL FIGURE LEGENDS**

**Supplemental figure 1: RAD51 forms elongated foci at sites of DNA damage**

A) RAD51 foci elongation (green) in HAP1, U2OS and HCT116 cells resolved by super-resolution. Scale bar, 2 μm. A max projection is shown. B) Immunofluorescence staining of RAD51 (green) puncta and elongated foci and γH2AX foci (magenta) in RPE-1 wild-type cells resolved by super-resolution. Cells were irradiated with 4 Gy and fixed 6 and 16 hours later. Scale bar, 5 μm. C) Consecutive z-slices from the 2 insets in (B) showing RAD51 elongated foci (green) emerging from a γH2AX focus (magenta) in RPE-1 cells. D) Extended gallery of example images of RAD51 elongated foci emanating from a γH2AX core. Scale bar, 1 μm. E) Immunofluorescence staining of RAD51 (magenta) foci combined with EdU (cyan) in RPE-1 wild-type cells. EdU was added 15 min before fixation to identify elongated RAD51 structures in S-phase cells by super-resolution. Cells were irradiated with 4 Gy and fixed 16 hours later.

**Supplemental figure 2: RAD51 forms elongated foci at sites of DNA damage**

A) Average amount of RAD51 foci (puncta (grey dots) and elongated foci (blue dots)) at 6 and 16 hours after IR (4 Gy). Data were obtained using Ilastik-based analysis (N=3). Each dot represents one cell and data from each replicate is depicted with an distinct symbol. Data are from the same experiments as in Fig.2C. B-D) Ilastik-based analysis of the longest RAD51 path (B) (6 vs 16 hours; p = 0,1617) and RAD51 area (C) (6 vs 16 hours; p = 0,0491) or RAD51 focus circularity (D) (6 vs 16 hours: p = 0,3114) of individual RAD51 foci in RPE-1 wild-type cells at 6 and 16 hours after irradiation (4 Gy). Each dot represents a single RAD51 focus. Data were obtained through Ilastik (N=3, *n = >*100 foci per replicate). Centre line, median; box limits, 25th and 75th centiles. Statistical significance is calculated by unpaired t-test using Prism. E) Example images (left) and quantification (right graph) of sister locus distances based on DHFR-FISH staining in G2 RPE-1 cells for which at least two DHFR foci were visible. DNA was stained by using DAPI. The red FISH signal was from the DHFR FISH probe. Scale bar, 5 μm. *n* ≥ 53 measurements.

**Supplemental figure 3: RAD54L is required for the resolution of RAD51 elongated foci**

A) Western blot confirming two independent RAD54L knock clones in HAP1 (HAP1ΔRAD54L). B) Western blot confirming two independent RAD54L knock clones in RPE-1 HALO-53BP1 (RPE-1 HALO-53BP1 ΔRAD54L). C) Clonogenic outgrowth of HAP1 wild-type and two monoclonal ΔRAD54L clones (green) in response to increasing doses of IR. Data presented as mean ± SEM (N=3). D) IncuCyte-based assay for caspase-3/7 activity. HAP1 wild-type and HAP1 ΔRAD54L 2.14 cells were irradiated (2 Gy) in the presence of a caspase-3/7 apoptosis assay reagent. Green fluorescence from the apoptosis assay reagent divided by the total confluence was used to estimate apoptosis for over time (N=3). E) Ilastik-based analysis of RAD51 focus circularity of individual RAD51 foci in wild-type and ΔRAD54L RPE-1 HALO-53BP1 cells at 6, 16 hours after irradiation (4 Gy). Each dot represents an individual RAD51 focus. Data are presented with individual measurements depicted with an different symbol per replicate (N=3, *n* > 700 foci examined per replicate). Box plots representing: center line, median; box limits, 25th and 75th percentiles. (WT vs. ΔRAD54L at 6 hours: p = 0. 0,3302; WT vs. ΔRAD54L at 16 hours: p = 0.056). Statistical significance was assessed by two-way ANOVA with Šídák's multiple comparisons test. F) Average amount of RAD51 foci (puncta (grey dots) and elongated foci (blue dots)) at 6 and 16 hours after IR (4 Gy). Data were obtained through Ilastik (N=3). Each dot represents one cell and data from each replicate is depicted with a distinct symbol. Data are from the same experiments as in Fig.3D. G) Super-resolution images of RPE-1 HALO-53BP1 cells. Cells were exposed to irradiation (4 Gy) and fixed 16 hours later and immunostained with RAD51 (green) and γH2AX (magenta). From top to bottom: I-shaped, Y-shaped or V-shaped RAD51 elongated foci emerging from a γH2AX focus. Scale bars, 0,5 μm.

**Supplemental figure 4: Cohesin, not CTCF, controls RAD51-mediated repair**

A) Scheme used for experiments performed with the HCT116 RAD21-mAIDmClover cells. Cells were either treated with DMSO (+cohesin) or 5-Ph-IAA (-cohesin) 1 hour before irradiation. Cells were fixed for immunofluorescence or harvested for western blot 6 or 16 hours after irradiation. Western blot showing near complete degradation of RAD21 after treatment with 5-Ph-IAA (-cohesin) for 7 hours in HCT116 RAD21-mAID-mClover cells. Western blot samples were harvested 6 hours after irradiation (4 Gy). B) Stills from a live-cell imaging experiment using asynchronously growing HCT116 RAD21-mAID-mClover cells with DNA visualized using sirDNA. Cells were either treated with DMSO (+cohesin) or 5-Ph-IAA (-cohesin), and depletion of RAD21 (-cohesin) was determined based on mClover fluorescence (first top/bottom panel). Cells were imaged every 5 min and their progression into mitosis was assessed (right panels). Scale bar 2 μm. C-D) HCT116 RAD21-mAID-mClover cells treated with DMSO (+cohesin) and 5-Ph-IAA (-cohesin) were left unirradiated (C) or irradiated with 2 Gy (D) and subsequently imaged for 24 hours. Relative mitotic entry of asynchronous cells is shown (N=2). E) Average amount of RAD51 foci (puncta (grey dots) and elongated foci (blue dots)) at 6 and 16 hours after IR (4 Gy). Data were obtained through Ilastik (N=3). Each dot represents one cell and data from each replicate is depicted with a distinct symbol. Data are from the same experiments as in Fig.5E. F) Ilastik-based analysis of RAD51 focus circularity of individual RAD51 foci in HCT116 RAD21-mAID-mClover cells treated with DMSO (+cohesin) and 5-Ph-IAA (-cohesin) at 6, 16 hours after irradiation (4 Gy). Each dot represents an individual RAD51 focus. Data are presented with individual measurements depicted with a different symbol per replicate (N=3, *n* > 700 foci examined per replicate). Box plots representing: center line, median; box limits, 25th and 75th percentiles. G) Western blot showing near complete degradation of CTCF after treatment with 5-Ph-IAA (CTCF) for 7 hours in HCT116 CTCF-mAID-mClover cells. Western blot samples were harvested 6 hours after irradiation (4 Gy). H) Quantification of the amount of RAD51 foci 6 and 16 hours after irradiation (4 Gy) in HCT116 CTCF-mAID-mClover cells. CTCF was depleted by adding 5-Ph-IAA (-CTCF) 1 hour prior to irradiation. I) Ilastik-based analysis of the longest RAD51 path of individual RAD51 foci in HCT116 CTCF-mAID-mClover cells at 6 and 16 hours after irradiation (4 Gy). Data were obtained through Ilastik (N=2). Every dot represents a single RAD51 focus. Data are presented with individual measurements and box plots. Centre line, median; box limits, 25th and 75th percentiles. J) HCT116 CTCF-mAID-mClover cells treated with DMSO (+CTCF) and 5-PhIAA (-CTCF) were irradiated with 2 Gy (as in D) and subsequently imaged for 24 hours. Relative mitotic entry of asynchronous cells is shown. K) Ilastik analysis of RAD51 focus circularity of individual RAD51 foci in HCT116 RAD21-mAID-mClover cells treated with DMSO (+cohesin) and 5-Ph-IAA (-cohesin) combined with siRNA treatment (siNT or siRAD54L) at 6 (left) and 16 (right) hours after irradiation (4 Gy). Each dot represents an individual RAD51 focus. Data are presented with individual measurements depicted with an different symbol per replicate (N=3, *n* > 700 foci examined per replicate). Box plots representing: center line, median; box limits, 25th and 75th percentiles. Statistical significance was assessed by two-way ANOVA with Turkey’s multiple comparisons test and p-values are shown for each comparison.

**Supplemental figure 5: Functionality of GFP-MND1**

A) Western blot analysis of HOP2, GFP and HSP90 in wild-type RPE1 HALO-53BP1, RPE1 HALO-53BP1 ΔMND1 and RPE1 HALO-53BP1 ΔMND1 expressing GFP-MND1 (ΔMND1^GFP-MND1^). HOP2 stabilization was assessed next to HSP90 as loading control and GFP for GFP-MND1 expression. B-C) Quantification of 53BP1 foci (B) and RAD51 foci (C) in RPE1 HALO-53BP1 wild-type (WT), MND1 knock-out (ΔMND1) or MND1 knock-out expressing MND1-GFP (ΔMND1^GFP-MND1^) without (0 Gy) or 8 hours after 4 Gy. D) Normalized IC50 of neocarzinostatin (NCS) in RPE1 HALO-53BP1 wild-type (WT), MND1 knock-out (ΔMND1) or MND1 knock-out expressing MND1-GFP (ΔMND1^GFP-MND1^). E) Mitotic entry 16 hours after either 0 Gy or 2 Gy in RPE1 HALO-53BP1 wild-type (WT), MND1 knock-out (ΔMND1) or MND1 knock-out expressing MND1-GFP (ΔMND1^GFP-MND1^).

**Supplemental figure 6: RAD51 elongated foci recruit MND1**

A-B) Overview (A) and randomly picked structures (B) of super-resolution images showing co-localization of GFP-MND1- (green) and RAD51-foci (magenta) in wild-type and ΔRAD54L RPE-1 HALO-53BP1 GFP-MND1 cells 16 hours after irradiation (4 Gy). Scale bar, 4 μm (A). Insets, Scale bar, 1 μm (B). C) Example plot of sphericity versus longest axis of GFP-MND1 foci measured in 3D in a single cell at 6 or 24 hours after irradiation (4 Gy). Morphologies of actual foci are presented in the graph. Adjacent graphs represent quantification of sphericity (top) and longest axis (bottom) of GFP-MND1 foci measured across multiple cells. The higher the value for sphericity, the more spherical (puncta) a focus is, the lower the value, the more elongated the focus is. Data were obtained through Imaris. D) Zoom-ins from stills taken from a live-cell imaging experiment showing the assembly of different GFP-MND1 (green) elongated foci emanating from 53BP1 foci in a wild-type RPE-1 HALO-53BP1 (magenta) RPA1-mScarlet (cyan) GFP-MND1 (green) cell exposed to irradiation (4 Gy). E-F) Representative images of GFP-MND1 (green) puncta and elongated foci and γH2AX foci (magenta) in RPE-1 HALO-53BP1/GFP-MND1 cells obtained by super-resolution. Cells were irradiated with 4 Gy and fixed 16 hours later. Scale bar, 4 μm. Insets, Scale bar, 1 μm. G) Examples of wide-field images of RPE-1 HALO-53BP1/GFP-MND1 cells exposed to irradiation (4 Gy) and fixed 16 hours after. Cells were immunostained with anti-GFP (green), anti-BRCA1 or anti-BRCA2 (cyan) and DAPI to stain DNA.

**Supplemental figure 7: Elongated HALO-MND1 foci in S and G2 phase**

A) HALO-MND1 overexpression induced by doxycycline addition in RPE-1 wild-type cells and RPE-1 FUCCI cells. B) Example image of a G2-phase, RPE-1 FUCCI HALO-MND1 cell showing elongated foci 16 hours after irradiation (4 Gy). C) Field of view of a live-cell imaging movie of RPE-1 FUCCI HALO-MND1 cells at three cell cycle stages, Cell 1 = G2 (green), Cell 2 = S-phase (yellow) and Cell 3 = G1 phase (red). Image is taken 10 minutes before irradiation. D) Stills taken from a live-cell imaging experiment showing the formation of HALO-MND1 foci in S and G2 phase, but not in G1 cells exposed to irradiation (4 Gy). E) HALO-MND1 foci (magenta) induced by irradiation (4 Gy, 6 and 16 hours) in HCT116 RAD21-mAID-mClover HALO-MND1 cells with (+cohesin) or without (-cohesin). Cells were treated with 5-Ph-IAA to deplete RAD21 (-cohesin) or DMSO (+cohesin) 1 hour before irradiation. Images were acquired on a confocal microscope. F) Stills from a live-cell imaging experiment using HCT116 RAD21-mAID-mClover HALO-MND1 (grey). Cells treated with 5-Ph-IAA to deplete RAD21 (-cohesin) or DMSO (+cohesin) 1 hour before irradiation (4 Gy). Clear HALO-MND1 foci accumulation is visible. G) Quantifications of HALO-MND1 foci per cell from live-cell movies (N=3).

**Supplemental figure 8: Bridging events in homology search**

A) Super-resolution images of RPE-1 HALO-53BP1 cells. Cells were exposed to irradiation (4 Gy), fixed after 16 hours and immunostained with anti-RAD51 (green) and anti-γH2AX (magenta). A bridging event connecting 2 separate γH2AX foci is shown. Scale bar, 0,5 μm. B) Zoom-in of a still from a live-cell imaging experiment using wild-type RPE-1 HALO-53BP1 (magenta) RPA1-mScarlet (cyan) GFP-MND1 (green) cells treated with NCS depicting a bridging MND1 elongated focus between two 53BP1 and RPA1 foci. C) Stills from a live-cell imaging experiment using wild-type RPE-1 HALO-53BP1 (magenta) RPA1-mScarlet (cyan) GFP-MND1 (green) cells treated with NCS depicting a bridging MND1 elongated focus between two 53BP1 and RPA1 foci. (Movie starts 5 hours after NCS treatment, imaged every 10 min). Scale bar, 0.5 μm. D) Super-resolution of RAD51- (green) and RPA2-foci (magenta) in RPE-1 HALO-53BP1, showing two connected RPA2 dots along the RAD51 elongated foci (16 hours, 4 Gy). Scale bar, 0.5 μm. E) Super-resolution of RAD51- (green) and RPA2-foci (magenta) in RPE-1 HALO-53BP1 cells (16 hours, 4 Gy) highlighting the connected dots along the elongated RAD51 foci. F) Two hypothetical models for foci bridging by elongated RAD51/MND1 foci. A RAD51 elongated focus (green) forms from the ‘core’ of the DSB, which is covered by RPA2 (magenta). 1) There are alternating patches of RAD51/RPA2, where not all the RPA2 is replaced for RAD51. (2) The broken end dynamically explores the nuclear environment, navigating through the crowded nucleus to seek its homologous sequence. Upon capturing a sequence that appears homologous, a capture event occurs. This homology sampling can occur along an intersegmental stretch of DNA, possibly multiple times, allowing RPA2/53BP1 to bind. If the sequence is heterologous, the elongated foci release it and continues searching. If the sequence is homologous, consecutive repair processes ensue. G-H) Stills from a live-cell imaging experiment using wild-type RPE-1 HALO-53BP1 (magenta) RPA1-mScarlet (data not shown) GFP-MND1 (green) cells depicting a bridging MND1 elongated focus between two 53BP1 foci. A single 53BP1 focus appears to split apart and the intensity of the original focus is redistributed between the two resulting dots. Scale bar, 0.5 μm.
