# Supplementary material for: The dynamics of RAD51 foci formation and elongation in living human cells
Source: Nucleic Acids Res. 2026 Sep 11;54(17):gkag863. doi: 10.1093/nar/gkag863 (PMC13563089; doi:10.1093/nar/gkag863)
Supplement: Supplementary file 1 [file gkag863_supplemental_files.zip › Supplementary video legends.docx]

**Supplementary video legends**

Suppl.Video1.

RPE-1 HALO-53BP1 (magenta) GFP-MND1 (green) cell imaged every 10 min showing the changes in morphology of multiple MND1 foci from formation to elongation and disassembly. Z projection is applied.

Suppl.Video2.

Cropped GFP-MND1 focus (white) from time-lapse experiment shown in Suppl.Video1. Images acquired every 10 minutes (Z-projection). The time-lapse captures the dynamic cycle of individual MND1 foci. Following macro processing, a binary mask (red) is applied to the segmented object and used to quantify the length of elongated MND1 foci.

Suppl.Video3.

RPE-1 HALO-53BP1 (magenta) GFP-MND1 (green) cell imaged every 10 min showing the the changes in morphology of various MND1 foci that are associated with connecting/splitting events of 53BP1 foci. Z projection is applied.

Suppl.Video4.

Cropped GFP-MND1 focus (green) from time-lapse images of RPE-1 HALO-53BP1 GFP-MND1 cells acquired every 10 minutes (Z-projection). The time-lapse captures splitting event of an 53BP1 focus (magenta) in connection with a GFP-MND1-positive elongated focus.

Suppl.Video5.

Cropped GFP-MND1 focus (green) from time-lapse images of RPE-1 HALO-53BP1 GFP-MND1 cells acquired every 10 minutes (Z-projection). The time-lapse shows a GFP-MND1-positive elongated focus that connects two 53BP1 foci (magenta).
